# T-BET and EOMES sustain mature human NK cell identity and antitumor function

**DOI:** 10.1172/JCI162530

**Published:** 2023-07-03

**Authors:** Pamela Wong, Jennifer A. Foltz, Lily Chang, Carly C. Neal, Tony Yao, Celia C. Cubitt, Jennifer Tran, Samantha Kersting-Schadek, Sathvik Palakurty, Natalia Jaeger, David A. Russler-Germain, Nancy D. Marin, Margery Gang, Julia A. Wagner, Alice Y. Zhou, Miriam T. Jacobs, Mark Foster, Timothy Schappe, Lynne Marsala, Ethan McClain, Patrick Pence, Michelle Becker-Hapak, Bryan Fisk, Allegra A. Petti, Obi L. Griffith, Malachi Griffith, Melissa M. Berrien-Elliott, Todd A. Fehniger

**Affiliations:** 1 Department of Medicine, Division of Oncology,; 2 Department of Neurological Surgery, and; 3 Siteman Cancer Center, Washington University School of Medicine in St. Louis, St. Louis, Missouri, USA.

**Keywords:** Immunology, Innate immunity, NK cells

## Abstract

Since the T-box transcription factors (TFs) T-BET and EOMES are necessary for initiation of NK cell development, their ongoing requirement for mature NK cell homeostasis, function, and molecular programming remains unclear. To address this, T-BET and EOMES were deleted in unexpanded primary human NK cells using CRISPR/Cas9. Deleting these TFs compromised in vivo antitumor response of human NK cells. Mechanistically, T-BET and EOMES were required for normal NK cell proliferation and persistence in vivo. NK cells lacking T-BET and EOMES also exhibited defective responses to cytokine stimulation. Single-cell RNA-Seq revealed a specific T-box transcriptional program in human NK cells, which was rapidly lost following T-BET and EOMES deletion. Further, T-BET– and EOMES-deleted CD56^bright^ NK cells acquired an innate lymphoid cell precursor–like (ILCP-like) profile with increased expression of the ILC-3–associated TFs *RORC* and *AHR*, revealing a role for T-box TFs in maintaining mature NK cell phenotypes and an unexpected role of suppressing alternative ILC lineages. Our study reveals the critical importance of sustained EOMES and T-BET expression to orchestrate mature NK cell function and identity.

## Introduction

Natural killer (NK) cells are innate lymphoid cells important for responses against pathogens and malignant cells. They direct the immune response through production of cytokines and directly kill diseased target cells via cytotoxic granules and death receptors (1, 2). In recent years, NK cell therapies have emerged as a promising option for treating cancers because of their low toxicity profile and their potent ability to drive antitumor responses (3–5). NK cell products can be sourced from peripheral blood as well as differentiated from cord blood or induced pluripotent stem cells (6–8). Additional strategies to enhance NK cell function, such as inducing memory-like NK differentiation with cytokines (3, 5, 9–11), introducing tumor-targeting chimeric antigen receptors (12), or using NK cells in combination with tumor-targeting antibodies or inhibitory checkpoint blockades, are now being tested in multiple clinical trials with promising results (6). Thus, understanding the fundamentals of mature NK cell biology will inform the nascent field of NK cell immunotherapy.

Studies in mice have shown that the T-box transcription factors (TFs) EOMES and T-BET are required for initiation of NK cell development, and their expressions persist in mature murine and human NK cells after development (13, 14). Constitutive, NK cell–specific knockout of EOMES or T-BET occurring at early stages of development results in a deficiency of mature NK cells (15, 16). In addition, T-BET also regulates mouse NK cell trafficking out of the bone marrow (17). However, because these models lack mature NK cells, the study of EOMES and T-BET in fully mature NK cells has not been feasible, and thus their ongoing importance to NK cell biology during maturity remains an open question in the field. Use of a tamoxifen-inducible NK cell–specific Cre mouse model revealed that EOMES was required for regulation of homeostasis and function of mature mouse NK cells, in a murine stage–specific fashion (18). Consistent with this, EOMES deletion in murine NK cells also impacted NK cell homeostatic turnover (19). Thus, while data related to EOMES and T-BET deficiency are emerging from inducible, conditional mouse models, our understanding of the importance of T-box TFs for mature human NK cell programs remains limited.

Most mechanistic studies of TFs in human NK cells to date have been performed in cell lines, NK cells differentiated in vitro from CD34^+^-derived hematopoietic stem cells, or NK cells that have been expanded ex vivo (20–22). While these models provide a starting point for study of mature human NK cell biology, these approaches do not recapitulate normal NK cell physiology. Recently, a single case of a patient with inherited T-BET deficiency was reported, with the patient exhibiting a defect in the NK cell compartment, and a separate study overexpressed EOMES and T-BET in NK progenitor cells, thereby promoting NK cell differentiation (20, 23). While these studies suggest that EOMES and T-BET play a role in human NK cell development, evaluation of EOMES or T-BET genetic loss of function in mature human NK cells has not been reported. Further, since EOMES and T-BET have similar DNA binding motifs and may play redundant roles, examination of mature NK cells with combined EOMES and T-BET deficiency is needed to address this gap in our knowledge and elucidate T-box TFs’ contribution to maintain NK cell molecular programs.

To address the importance of T-box TFs and their regulation of mature NK cell programs, we used CRISPR/Cas9 to genetically delete EOMES and T-BET expression in unexpanded, primary human NK cells. We hypothesized that beyond their roles in regulating NK development, EOMES and T-BET are critical for maintaining the NK cell functional programs that define NK cells, including proliferation, survival, cytotoxicity, and cytokine production. To define molecular mechanisms, we also used single-cell RNA-Seq and assay for transposase-accessible chromatin using sequencing (ATAC-Seq) to uncover key transcriptomic and chromatin changes in mature human NK cells with deletion of EOMES and T-BET. These findings revealed a profound dependency on T-box TFs to maintain mature human NK cell function and identity.

## Results

### CRISPR editing deletes T-BET and EOMES in unexpanded primary human NK cells.

Genetic manipulation of primary human NK cells has been a challenge in the field, limiting our ability to use loss or gain of function to mechanistically understand human NK cell biology. Peripheral blood NK cells were freshly isolated from healthy donors, purified to more than 95% by negative enrichment, cultured in low-dose IL-15 (required for NK cell survival), and electroporated with Cas9 mRNA along with sgRNA targeting *EOMES* or *TBX21* using the MaxCyte GT system (9). This approach achieved consistent deletion of EOMES and T-BET protein expression in both the CD56^bright^ and CD56^dim^ NK cell subsets without the need to expand them with high-dose cytokines or feeder cells (Supplemental Figure 1; supplemental material available online with this article; https://doi.org/10.1172/JCI162530DS1).

**Figure 1:**
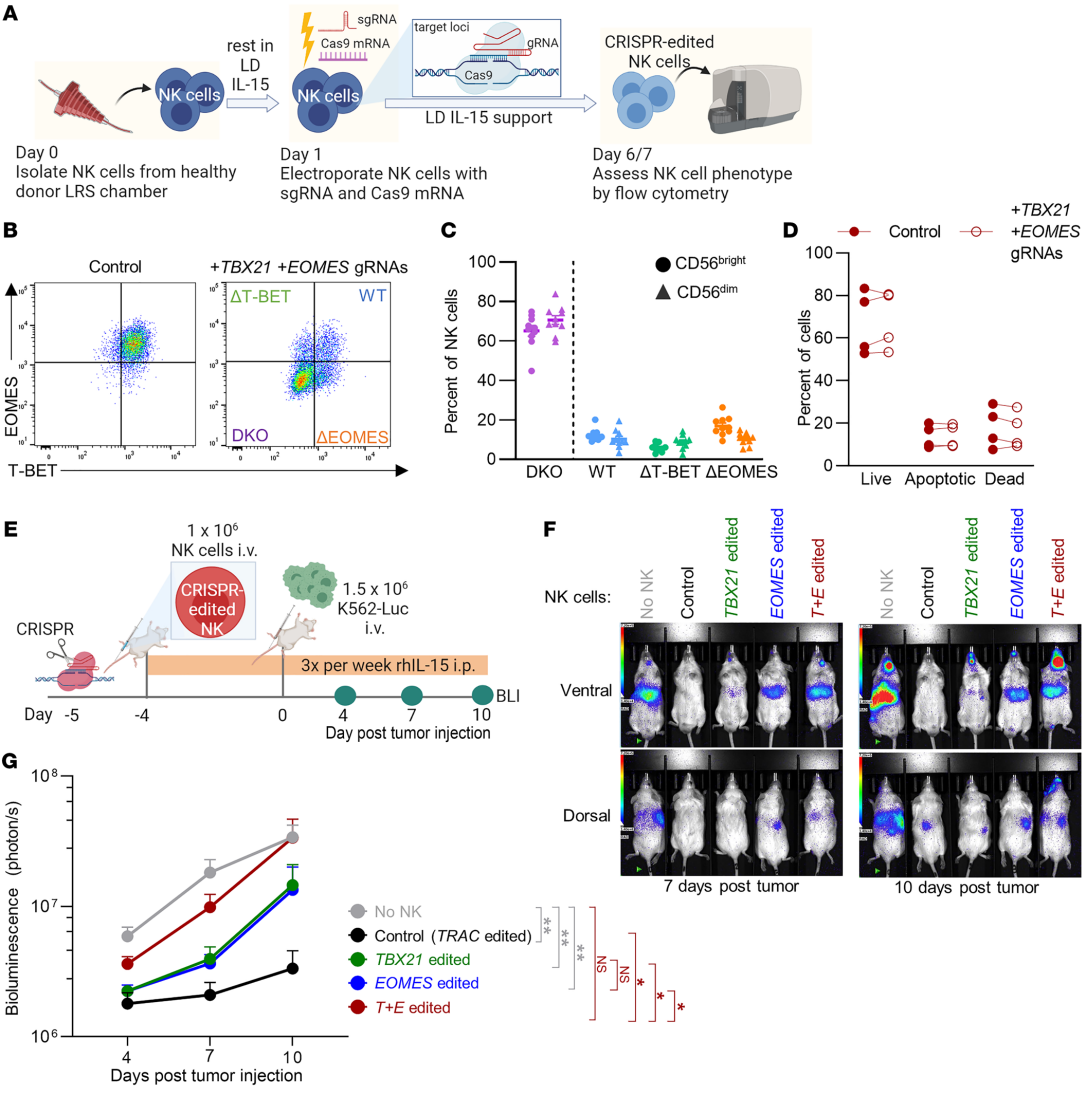
EOMES and T-BET are required for optimal tumor control in vivo. (**A**) NK cells were subjected to electroporation to deliver Cas9 mRNA and sgRNAs. NK cells were cultured in low-dose (LD) IL-15, and on day 6/7, T-BET and EOMES protein expression was assessed by flow cytometry. LRS, leukocyte reduction system. (**B**) Representative flow plot of EOMES and T-BET protein expression in control NK cells and NK cells targeted simultaneously with *TBX21* and *EOMES* gRNAs (*T+E* edited). (**C**) Summary data of DKO efficiency in *T+E* edited NK cells. *n* = 10 donors, 9 independent experiments. (**D**) *T+E* edited NK cells were cultured in LD IL-15, then harvested on day 6/7, stained for annexin V and 7-aminoactinomycin D, and analyzed by flow cytometry. *n* = 4 donors, 4 independent experiments. (**E**) NK cells from either *TRAC* gRNA–edited (control), *TBX21*-edited, *EOMES*-edited, or *T+E* edited group were injected i.v. into NSG mice. Four days later, mice were challenged with luciferase-expressing K562 tumor cells. Injections of rhIL-15 i.p. were performed 3 times a week to support the human NK cells. (**F**) Representative bioluminescent imaging (BLI) of tumor burden in NSG mice that received no NK, control NK, or TBX21- and EOMES-edited NK cells. (**G**) Summary data of tumor burden measured by BLI. Two outliers in the control NK group were identified by ROUT (81) (*Q* = 0.1%) and excluded from the analysis. *n* = 6–9 mice each group, from 5 donors, 5 independent experiments. Data were compared using 2-way ANOVA in **C** and **D**, and mixed-effects analysis with Holm-Šidák multiple-comparison test in **G**. *P < 0.05, **P < 0.01.

Since T-BET and EOMES have highly homologous DNA binding domains, deletion of one could result in compensation by the other (24, 25). To address their redundancy, both EOMES and T-BET were CRISPR-edited by simultaneous electroporation of *TBX21* and *EOMES* gRNAs, which successfully abrogated both EOMES and T-BET protein expression to generate double-knockout (DKO) NK cells (Figure 1, A–C). To discern between all cells that received the CRISPR gRNAs and the subset that were deficient for T-BET and EOMES at the protein level, “*T+E* edited” and “DKO” will be used, respectively. We observed no significant changes in *T+E* edited NK cell survival in vitro, in comparison with control cells that were CRISPR-edited with *TRAC* gRNA used as control, indicating that the two T-box TFs are not absolutely required for NK cell survival (Figure 1D). This approach of simultaneous T-BET and EOMES deletion then allows investigation of the functional impact of naturally developed and mature primary human NK cells lacking these two T-box TFs.

### EOMES and T-BET deletion does not affect short-term in vitro killing but impairs long-term in vitro killing.

One hallmark of NK cell function is their ability to eliminate MHC class I–deficient tumor cells (1, 2). To assess the impact of T-box TF deficiency on NK cells’ cytotoxic ability in a short-term assay, we sorted CD56^bright^ and CD56^dim^ NK cells 5 days after CRISPR editing, rested them overnight in low-dose IL-15, and then cocultured them with the MHC-I–deficient cell line K562 for 4–6 hours. In this short-term killing assay, there was no significant effect of T-box TF deficiency on NK cells’ ability to kill K562 targets (Supplemental Figure 2A). This was expected because of the presence of granzyme B and perforin protein within preformed granules in the NK cells that result in immediate cytotoxicity. To test the effect of T-box TF deficiency in a longer-term in vitro cytotoxicity assay, we used the IncuCyte Imaging System (Sartorius) to monitor NK cell control of the ovarian cancer cell line SKOV-3 over the course of 6 days (Supplemental Figure 2B). We similarly observed no difference between control NK cells and *T+E* edited NK cells at early time points, but over the course of 6 days, *T+E* edited NK cells were unable to control SKOV-3 cells as effectively as control NK cells (Supplemental Figure 2B). This is consistent with the need to replenish cytotoxic effector proteins after initial preformed granules are depleted.

### EOMES and T-BET are required for tumor control in vivo.

To assess the impact of T-box TF deficiency on NK cells’ ability to control tumor in vivo, we engrafted *TRAC* gRNA/CRISPR–edited (control NK), *TBX21-*edited, *EOMES*-edited, or *T+E* edited NK cells into NOD-*scid* IL2Rg^null^ (NSG) mice, which lack T, B, or NK cells and thus allow for xenograft of human cells (Figure 1E) (26). Recombinant human (rh) IL-15 was administered 3 times per week to support human NK cell survival. The NK cells were engrafted and allowed 4 days for T-BET and EOMES protein to be downregulated in vivo. The mice were then challenged with MHC-I–deficient K562-luciferase (K562-luc) tumor cells. Mice that received control NK cells had minimal bioluminescent imaging signals on day 7 and day 10 after tumor challenge, while mice that received *TBX21*- or *EOMES*-single-edited or *T+E* edited NK cells had significantly higher tumor burden than those that received control NK cells (Figure 1, F and G), indicating reduced NK cell antitumor response in the absence of T-BET and/or EOMES. While mice that received *TBX21*- or *EOMES*-single*-*edited NK cells still had significantly reduced tumor burden compared with mice that received no NK cells, mice that received *T+E* edited NK cells had an average tumor burden that was not significantly different from that of mice that did not receive NK cells (Figure 1G).

### EOMES and T-BET are required for NK cell persistence and proliferation in vivo.

**Figure 2:**
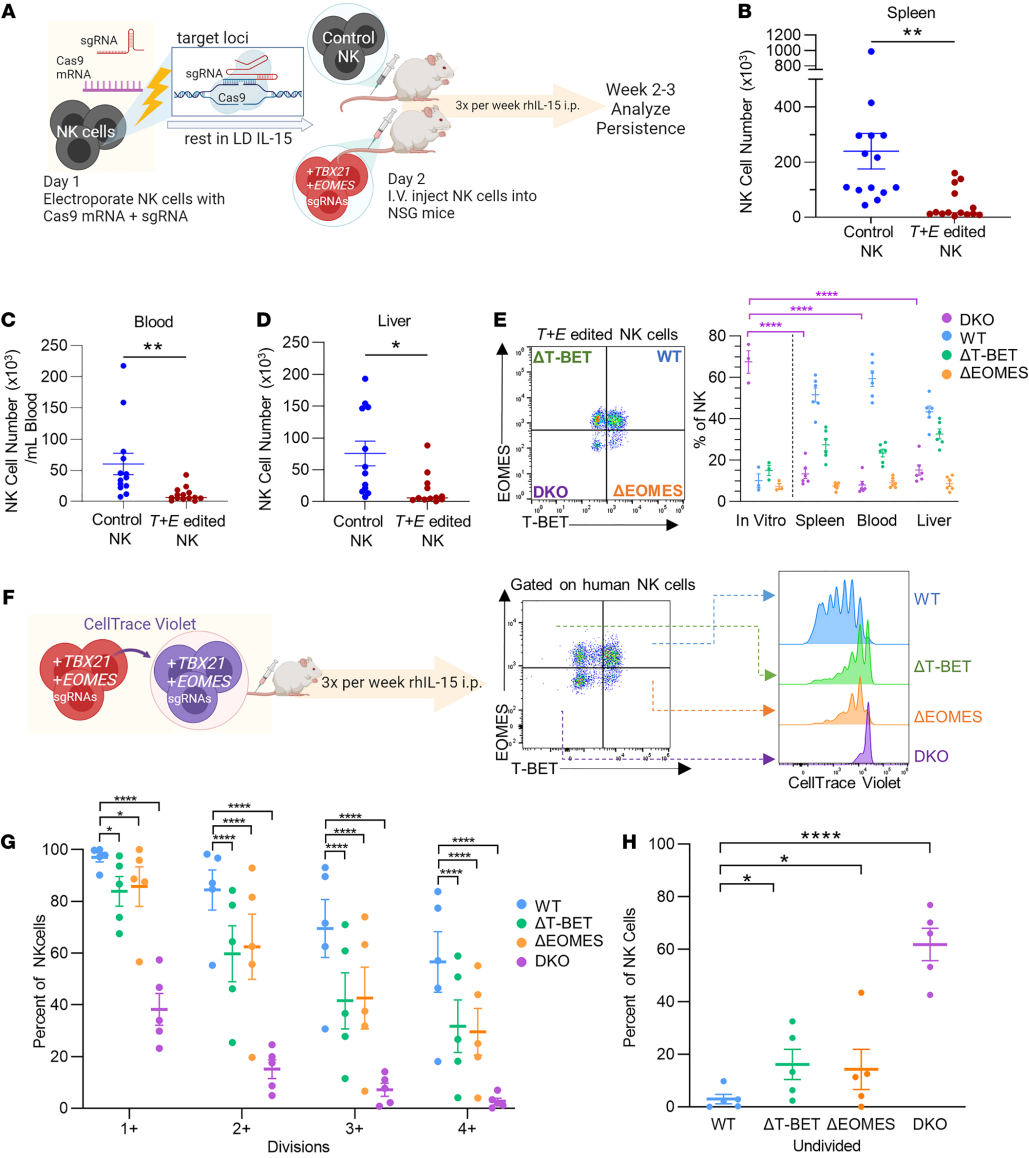
EOMES and T-BET are required for NK cell persistence and proliferation in vivo. (**A**) Experimental schema for **B**–**E**. (**B**–**D**) Summary data of NK cell numbers recovered from indicated tissues of NSG mice that received control NK cells or *T+E* edited NK cells. *n* = 12–15 mice per condition, 6 donors, 5 independent experiments. (**E**) WT (T-BET^+^EOMES^+^), ΔT-BET (T-BET^–^EOMES^+^), ΔEOMES (T-BET^+^EOMES^–^), and DKO (T-BET^–^EOMES^–^) cells were identified by flow cytometry. Left: Representative flow plot. Right: Summary data of the percentage of each population within the NK compartment of indicated tissues of mice that received *T+E* edited NK cells. In vitro percentages were assessed about 1 week after electroporation. *n* = 3 donors, 3 independent experiments. (**F**) Schema for proliferation study. *T+E* edited NK cells were labeled with CellTrace Violet (CTV) dye before injection into NSG mice. (**G** and **H**) 1.5–2 weeks later, percentages of NK cells that had undergone the indicated number of divisions (**G**) and those that had not divided (**H**) were assessed by CTV dye dilution by flow cytometry. *n* = 3 donors, 5 mice, 3 independent experiments. Data were compared using unpaired *t* test in **B**–**D** and 2-way ANOVA with Holm-Šidák multiple-comparison test in **E**, **G**, and **H**. *P < 0.05, **P < 0.01, ****P < 0.0001.

We hypothesized that the significant defect in tumor control by *T+E* edited NK cells was due to reduced persistence or proliferation or decreased functionality of the DKO NK cells. To address these possibilities, the ability of NK cells to undergo homeostatic proliferation and persist in vivo without EOMES and T-BET was evaluated. *T+E* edited NK cells were engrafted into NSG mice, and 2–3 weeks later the number of NK cells in various tissues was determined (Figure 2A and Supplemental Figure 3A). Notably, on average at least 75% fewer *T+E* edited NK cells were recovered compared with control NK cells in all 3 tissues investigated: spleen, blood, and liver (Figure 2, B–D). Since *T+E* editing is not completely efficient, we expected the small fraction of T-BET^+^EOMES^+^ wild-type (WT) NK cells to have an expansion advantage in vivo over this time course. Consistent with this, intracellular flow cytometry staining revealed that the number of *T+E* edited NK cells that were deficient for both T-BET and EOMES (DKO) at the protein level was significantly reduced in frequency after in vivo proliferation for 2–3 weeks, in comparison with the in vitro day 7 expression (Figure 2E). While NK cells expressing WT levels of EOMES and T-BET within the *T+E* edited group were only a minority after 7 days in vitro, these WT NK cells became a majority of the NK cells recovered from mice that received *T+E* edited NK cells when assessed after 2–3 weeks (Figure 2E), providing further evidence that the DKO NK cells had a competitive disadvantage.

Since cell death was minimally impacted in *T+E* edited NK cells (Figure 1D), we hypothesized that EOMES and T-BET are required for NK cell homeostatic proliferation, and this mechanism explains the lower number of *T+E* edited NK cells recovered following engraftment into NSG mice. To assess in vivo proliferation, *T+E* edited NK cells were labeled with CellTrace Violet dye and transferred into NSG mice, and dye dilution was quantified by flow cytometry after 1.5–2 weeks (Figure 2F). The numbers of divisions that flow-gated T-BET– and EOMES-WT, single-KO (ΔT-BET and ΔEOMES), and DKO NK cells underwent were assessed. While single T-BET or EOMES deletion resulted in impaired proliferation, deleting both T-BET and EOMES profoundly reduced proliferation (Figure 2, G and H). In fact, a majority of DKO NK cells did not proliferate, in contrast to almost all WT NK cells having divided at least once by this time (Figure 2H). This proliferation defect observed in DKO NK cells is consistent with the low numbers of NK cells recovered and the increase in the frequency of WT NK cells in the NK compartment from mice that received *T+E* edited NK cells (Figure 2, A–E).

Since it was previously reported in a murine NK cell study that NK cell proliferation can be regulated differentially at different states of maturation (27), we further categorized the human NK cells harvested from the NSG mice into human maturation stages, CD57^–^NKG2A^+^, CD57^+^NKG2A^+^, and CD57^+^NKG2A^–^, and assessed the effect of EOMES and T-BET deficiency on the proliferation of these specific subsets (Supplemental Figure 3B). DKO of T-BET and EOMES significantly reduced proliferation of all 3 subsets, while single deletion of T-BET or EOMES only reduced proliferation in the more mature CD57^+^ subsets (Supplemental Figure 3B).

### EOMES and T-BET deletion impairs NK cell cytokine production.

**Figure 3:**
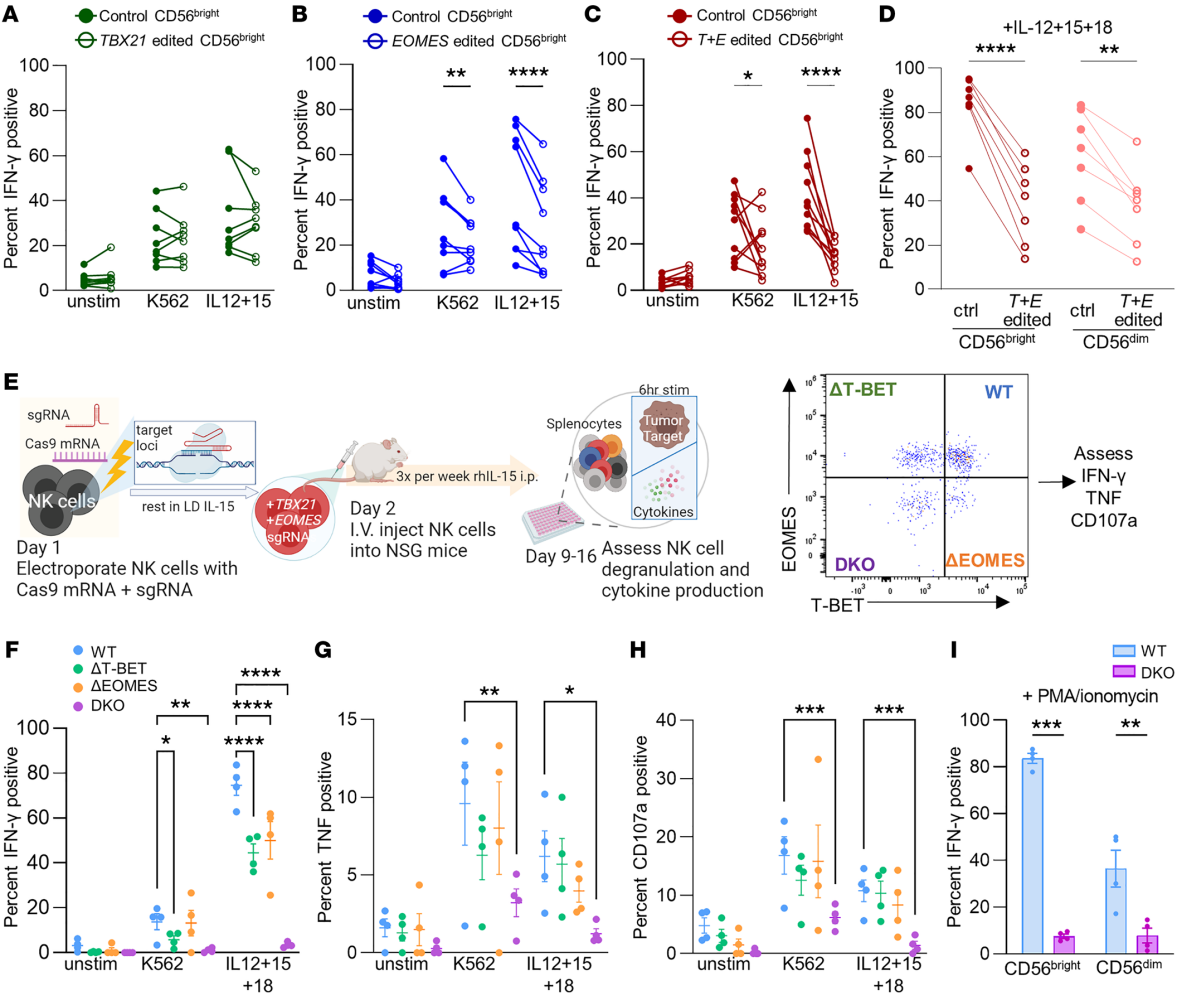
EOMES and T-BET deletion impairs NK cell cytokine response. (**A**–**D**) In vitro functional assessment. Day 6/7 after CRISPR electroporation, NK cells were stimulated with K562 and IL-12+15. Degranulation (CD107a) and IFN-γ production were quantified by intracellular flow staining. (**A**–**C**) Summary data of IFN-γ response by *TBX21*-edited (**A**), *EOMES-*edited (**B**), and *T+E* edited (**C**) CD56^bright^ NK cells. (**D**) Summary data of IFN-γ response control and *T+E* edited NK cells stimulated with IL-12+15+18. *n* = 7–10 donors, 4–7 independent experiments in **A**–**D**. (**E**) Schema of ex vivo functional assessment experiment. 1.5–2 weeks after NK cell injection, splenocytes were harvested and stimulated with K562, IL-12+15, and IL-12+15+18 for 6 hours. (**F**–**H**) Summary data of IFN-γ (**F**), TNF (**G**), and CD107a (**H**) in human NK cells within indicated T-BET/EOMES flow gate. *n* = 2 donors, 4 mice, 3 independent experiments. (**I**) 1.5 weeks after CRIPSR editing, in vitro–maintained NK cells were stimulated with PMA/ionomycin for 6 hours. IFN-γ production by NK cells by flow-gated CD56^bright^ and CD56^dim^ subsets is shown. *n* = 4 donors, 2 independent experiments. Data were compared by 2-way ANOVA with Holm-Šidák multiple-comparison test. *P < 0.05, **P < 0.01, ***P < 0.001, ****P < 0.0001.

We next assessed the functionality of NK cells after they were CRISPR-edited to abrogate T-BET and EOMES expression. Whereas nearly all in vivo–transferred human NK cells recovered were CD56^dim^, both CD56^bright^ and CD56^dim^ NK cell subsets were readily discernible in vitro (Supplemental Figure 1A and Supplemental Figure 3A). Based on the different functional characteristics of these subsets (28, 29), CD56^bright^ and CD56^dim^ NK cells were analyzed separately. CRISPR-edited NK cells were stimulated with K562 or cytokines (IL-12 and IL-15) and assessed for their ability to degranulate (surface CD107a) or produce immunomodulatory cytokines (IFN-γ and TNF), compared with control NK cells (Figure 3, A–D, and Supplemental Figure 4, A–F). *TBX21* editing alone did not significantly affect NK cell function in this assay (Figure 3A and Supplemental Figure 4, A and B). *EOMES* editing alone only reduced CD56^bright^, but not CD56^dim^, NK cell IFN-γ production after stimulation by cytokines or K562 (Figure 3B and Supplemental Figure 4, C and D). Simultaneous deletion of EOMES and T-BET led to marked reduction of IFN-γ and TNF production in both CD56^bright^ and CD56^dim^ NK cells, even when the potent stimulating cytokine combination of IL-12, IL-15, and IL-18 was used (Figure 3, C and D, and Supplemental Figure 4, E and F). Notably, degranulation in response to K562 cells was not affected by genetic deletion (Supplemental Figure 4, E and F).

To give the effect of T-BET and EOMES deletion more time to manifest prior to assessment of these functions, we engrafted NSG mice with NK cells CRISPR-edited with *TBX21* and *EOMES* gRNAs and 1.5–2 weeks later assessed functionality ex vivo with isolated splenocytes. Here, intracellular flow gating was used to separately analyze NK cells that were ΔT-BET or ΔEOMES single KO or DKO at the protein level (Figure 3, E–H). ΔT-BET and ΔEOMES single-KO NK cells had significantly impaired IFN-γ and TNF production in response to cytokine stimulation compared with WT NK cells (Figure 3, F and G). However, like in the in vitro setting, degranulation was not significantly impacted with single T-BET or EOMES deletion alone (Figure 3H). DKO of both T-BET and EOMES resulted in marked reduction of NK cell function ex vivo: DKO NK cells had minimal production of IFN-γ and TNF in response to the potent combined cytokine stimulation of IL-12 plus IL-15 plus IL-18 (Figure 3, F and G). In contrast to the in vitro setting, NK cells lacking both EOMES and T-BET also had marked impairment of K562-induced degranulation (Figure 3H). These data demonstrate that both EOMES and T-BET are critical for primary NK cell degranulation and cytokine production upon stimulation with cellular targets or proinflammatory cytokines.

We also tested whether T-BET– and EOMES-DKO NK cells could produce similar amounts of IFN-γ compared with control NK cells if we bypassed receptor signaling completely by using PMA and ionomycin to stimulate. Similar to responses following K562 or cytokine stimulation, PMA- and ionomycin-stimulated T-BET– and EOMES-DKO NK cells were able to produce only minimal IFN-γ (Figure 3I).

### EOMES and T-BET deletion impairs NK cell numbers and effector molecule expression at tumor sites.

**Figure 4:**
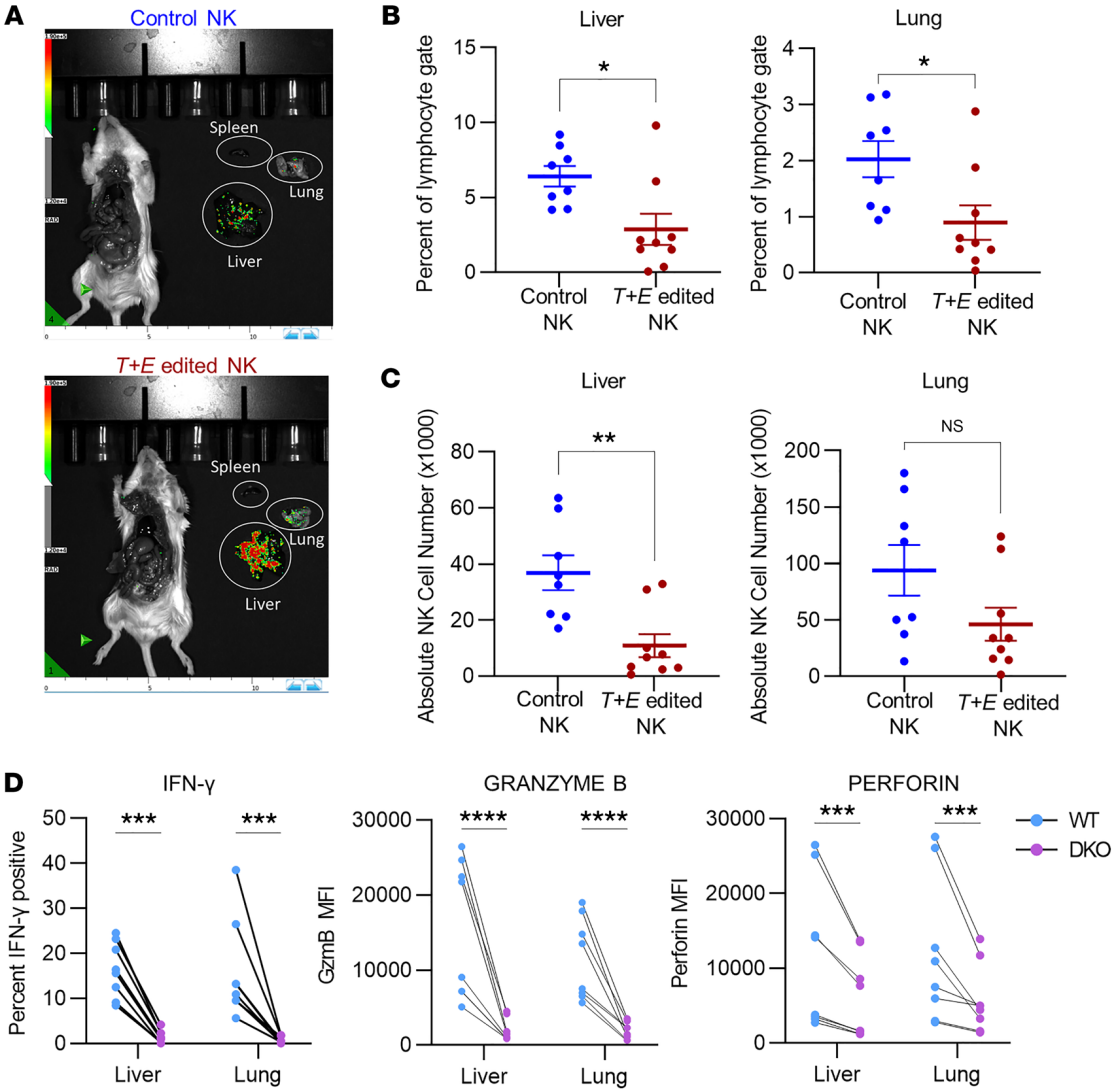
Deletion of EOMES and T-BET impairs NK cell numbers and effector molecule expression in K562 tumor–bearing mice. NK cells from either *TRAC* gRNA–edited (control) NK group or *T+E* edited NK group were injected i.v. into NSG mice the day after electroporation, and i.p. injections of rhIL-15 were performed 3 times a week to support the human NK cells. Four days after NK injection, mice were challenged i.v. with 1.5 × 10^6^ ± 0.1 × 10^6^ luciferase-expressing K562 tumor cells. Three days after tumor injection, mice were imaged, and tumor-bearing tissues were harvested and assessed. (**A**) Representative BLI image of tumor signals. (**B** and **C**) Percent of lymphocyte gate (FSC by SSC) (**B**) and absolute NK cell number (**C**) in the livers and lungs of tumor-bearing mice. (**D**) Flow cytometry assessment of IFN-γ, granzyme B, and perforin expression in gated WT and DKO NK cells from the liver and lung of tumor-bearing mice that received *T+E* edited NK cells. Data were compared with Welch’s *t* test in **B** and **C**, and 2-way ANOVA with Holm-Šidák multiple-comparison test in **D**. *n* = 8–9 mice per group, 4 donors, 4 independent experiments. *P < 0.05, **P < 0.01, ***P < 0.001, ****P < 0.0001.

To assess the in vivo responses of EOMES- and T-BET–deficient NK cells, we followed the same experimental approach as in Figure 1E, but sacrificed K562-luc tumor–bearing mice 3 days after tumor injection to assess numbers and phenotypes of control versus T-BET– and EOMES-DKO NK cells (Figure 4). At this time point, while *T+E* edited NK cells were able to traffic to the tumor-infiltrated lung and liver, *T+E* edited NK cell percentages and absolute numbers were reduced in comparison with control NK cells (Figure 4, A–C). In addition, T-BET– and EOMES-DKO NK cells harvested from tumor-bearing organs had reduced IFN-γ, granzyme B, and perforin compared with WT NK cells (Figure 4D).

### IL-15 and IL-12 receptor signaling responses are impacted by lack of T-BET and EOMES.

**Figure 5:**
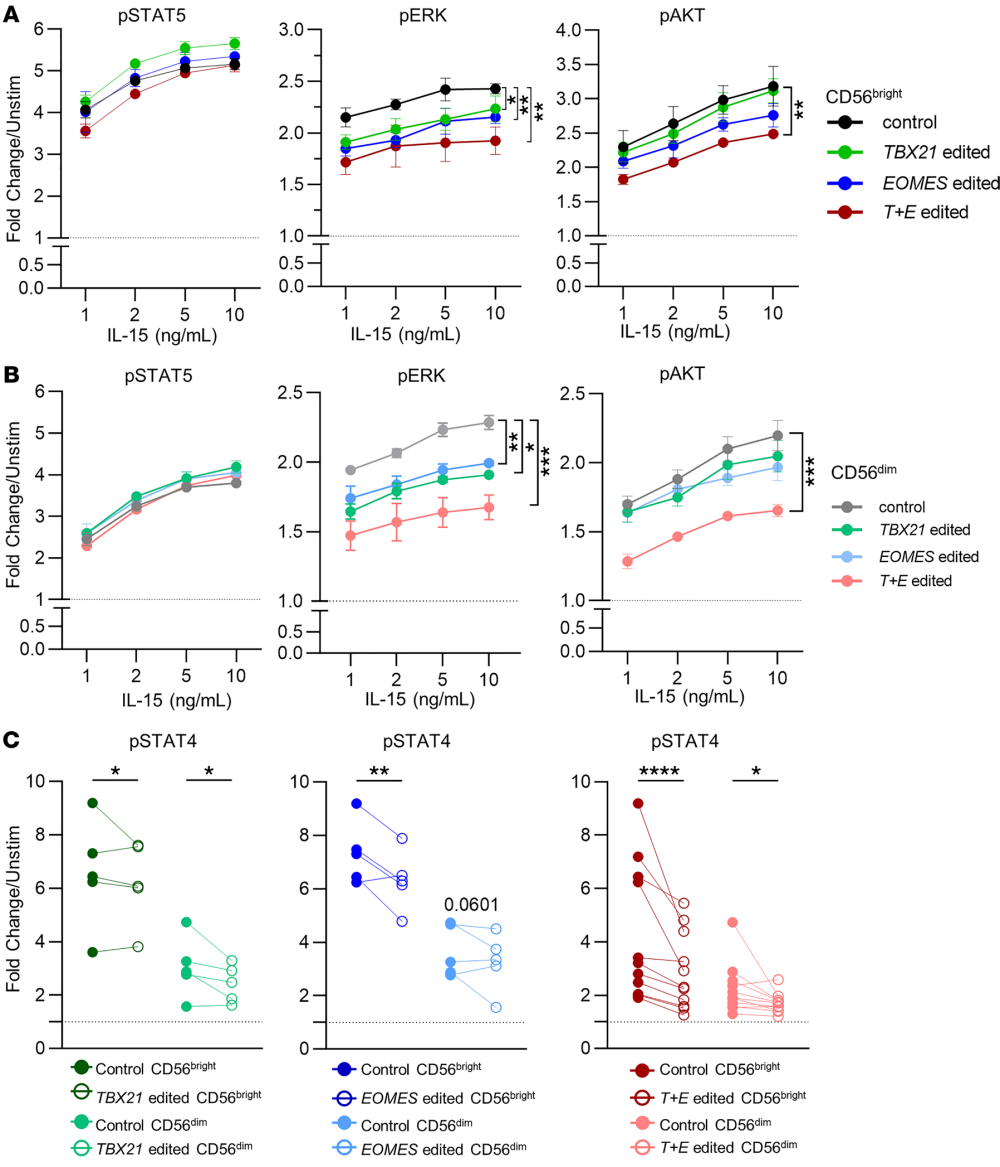
Deletion of EOMES and T-BET impairs phosphorylation of ERK and AKT downstream of IL-15 signaling. NK cells were rested in cytokine-free medium for 1 hour before stimulation with the indicated concentrations of IL-15. Phosphorylated STAT5 (p-STAT5) was assessed in cells stimulated for 15 minutes, while p-ERK and p-AKT were assessed at 1 hour. (**A** and **B**) Summary data of MFI fold change of CD56^bright^ (**A**) and CD56^dim^ (**B**) NK cells. *n* = 3 donors, 3 independent experiments. (**C**) Summary data of STAT4 phosphorylation upon IL-12 stimulation. p-STAT4 was assessed in cells stimulated with IL-12 for 1 hour. *n* = 5–8 donors, 3–6 independent experiments. Data were compared with 2-way ANOVA with Holm-Šidák multiple-comparison test. *P < 0.05, **P < 0.01, ***P < 0.001, ****P < 0.0001.

Multiple signaling pathways downstream of the IL-15 receptor (IL-15R) impact NK cell functions, including survival, proliferation, cytokine production, and cytotoxicity (30). We hypothesized that signaling downstream of cytokine receptors may be defective in NK cells that lack T-BET and EOMES, thereby explaining their proliferative and functional defects. The NK cell response to IL-15R stimulation was assessed by measurement of phosphorylation of signaling intermediate proteins in pathways downstream of IL-15R (STAT5, ERK1/2 [MAPK], and AKT pathways) by flow cytometry upon IL-15 stimulation (Figure 5, A and B) (30). In both CD56^bright^ and CD56^dim^ NK cells, phosphorylation of STAT5 was unaltered, even in NK cells that lacked both T-BET and EOMES, consistent with their intact survival. However, phosphorylated ERK (p-ERK) was reduced in *TBX21*- or *EOMES*-single-edited NK cells, and this defect was markedly more evident in *T+E* edited NK cells (Figure 5, A and B). p-AKT was only significantly decreased in *T+E* edited NK cells in both CD56^bright^ and CD56^dim^ subsets (Figure 5, A and B). The responsiveness of *TBX21*-, *EOMES*-, and *T+E* edited NK cells to IL-12R signaling was assessed by STAT4 phosphorylation (Figure 5C). p-STAT4 of CD56^bright^ NK cells was significantly impacted by deletion of one or both T-box TFs. p-STAT4 of CD56^dim^ NK cells followed the same trend, with statistically significant reduction in *TBX21*-edited and *T+E* edited NK cells, and a non-significant trend (*P* = 0.06) in *EOMES*-edited NK cells (Figure 5C).

Western blots of control, *TBX21-*edited, *EOMES*-edited, and *T+E* edited NK cell lysates show that total AKT, ERK1/2, and STAT4 proteins were not affected by T-BET or EOMES deletion, indicating that the observed differences in phosphorylated proteins were not due to decreases in total protein level of the specific signaling protein (Supplemental Figure 5, A and B; see complete unedited blots in the supplemental material). We also assessed the expression of the IL-15R subunit CD122 and found no significant differences between control and *T+E* edited NK cells maintained in vitro at the time point when the phosphorylation assay was performed 6 days after electroporation (Supplemental Figure 5C).

### EOMES and T-BET are required to sustain the NK cell transcriptional program.

As T-BET and EOMES primarily orchestrate gene transcription, we evaluated NK cell transcriptomes following T-BET and EOMES deletion. Single-cell RNA-Seq (scRNA-Seq) (10x Genomics) was performed on *TRAC*-edited (control) and *T+E* edited NK cells. CRISPR-edited NK cells from each donor were transferred into NSG mice, followed by rhIL-15 support 3 times per week for survival. After 1 week, splenocytes of the NSG mice were isolated, and human NK cells were purified by flow sorting (mCD45^–^hCD45^+^hCD3^–^hCD56^+^, >98% purity; 3 donors in vivo). To provide an in vitro comparison to account for in vivo phenotypic changes, and to allow analysis of both CD56^bright^ and CD56^dim^ NK cell subsets, CRISPR-edited NK cells were cultured for 1 week in vitro in parallel (5 donors total in vitro). In vitro and in vivo data sets were analyzed separately, contrasting control-edited and *T+E* edited NK cells (Supplemental Figure 6A). This approach revealed distinct clusters of NK cells in uniform manifold approximation and projection (UMAP) space (Supplemental Figure 6, B and C).

**Figure 6:**
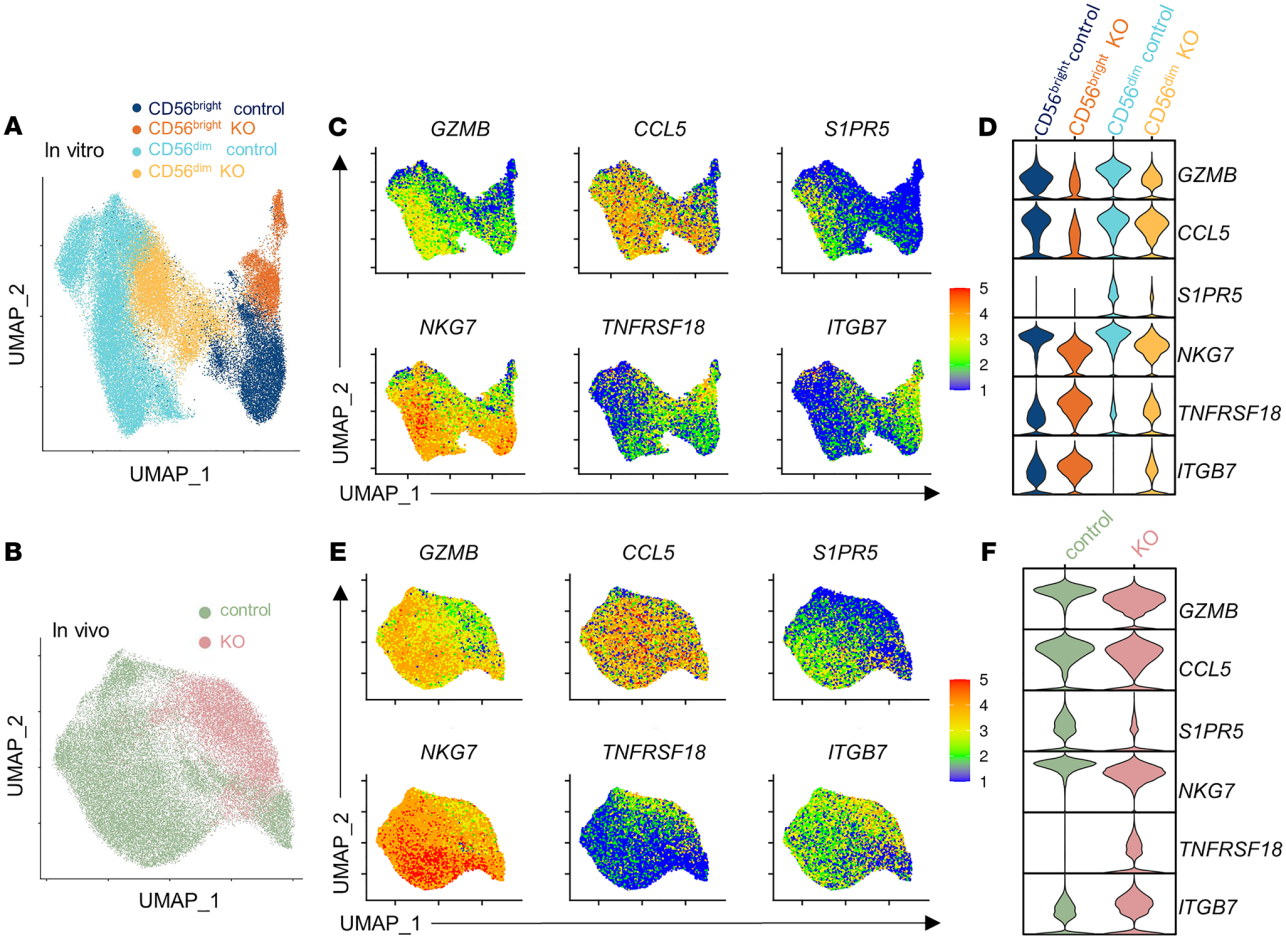
Single-cell RNA sequencing reveals transcriptional profile regulated by EOMES and T-BET in human NK cells. In vitro– and in vivo–maintained CRISPR-edited human NK cells were subjected to scRNA-Seq. Data shown are from pooled analysis of *n* = 3–5 donors from 3–5 independent experiments. (**A** and **B**) UMAP of NK cells maintained in vitro (**A**) and in vivo (**B**), with control and KO clusters identified (see Methods and Supplemental Figure 6). (**C** and **E**) Expression level of selected DEGs overlaid on UMAP. (**D** and **F**) Violin plots of expression of selected DEGs in control and KO clusters. Wilcoxon’s rank-sum test was used for differential analysis with a threshold of adjusted *P* value less than 0.05.

The single-cell sequencing approach allowed us to distinguish KO cells within the heterogeneous population of edited cells in the analysis. We first assigned in vitro clusters to be either CD56^bright^ or CD56^dim^ NK cells based on established scRNA-Seq expression patterns of markers for each subset (Supplemental Figure 6, B and C) (31, 32). Almost all NK cells recovered 1 week after transfer in vivo were CD56^dim^ NK cells, since all clusters expressed the CD56^dim^ subset marker *FCGR3A* (CD16) (Supplemental Figure 6C). While EOMES and T-BET proteins are expressed by all conventional NK cells, their transcript levels are known to be relatively low and difficult to detect because of limitations in existing scRNA-Seq technology. Thus, we used the sample origin proportion of each cluster to identify the cells that were most likely to be T-BET– and EOMES-KO, as those cells would be unique to *T+E* edited samples (Supplemental Figure 6, B and C, and see Methods). Notably, the majority of cells belonging to cycling clusters from both in vitro and in vivo originated from control samples, which is consistent with our experimental data demonstrating impaired proliferation of KO NK cells (Supplemental Figure 6, B and C, and Figure 2, F–H). Finally, we reclustered only the non-cycling control and KO clusters for visualization (Figure 6).

**Figure 7:**
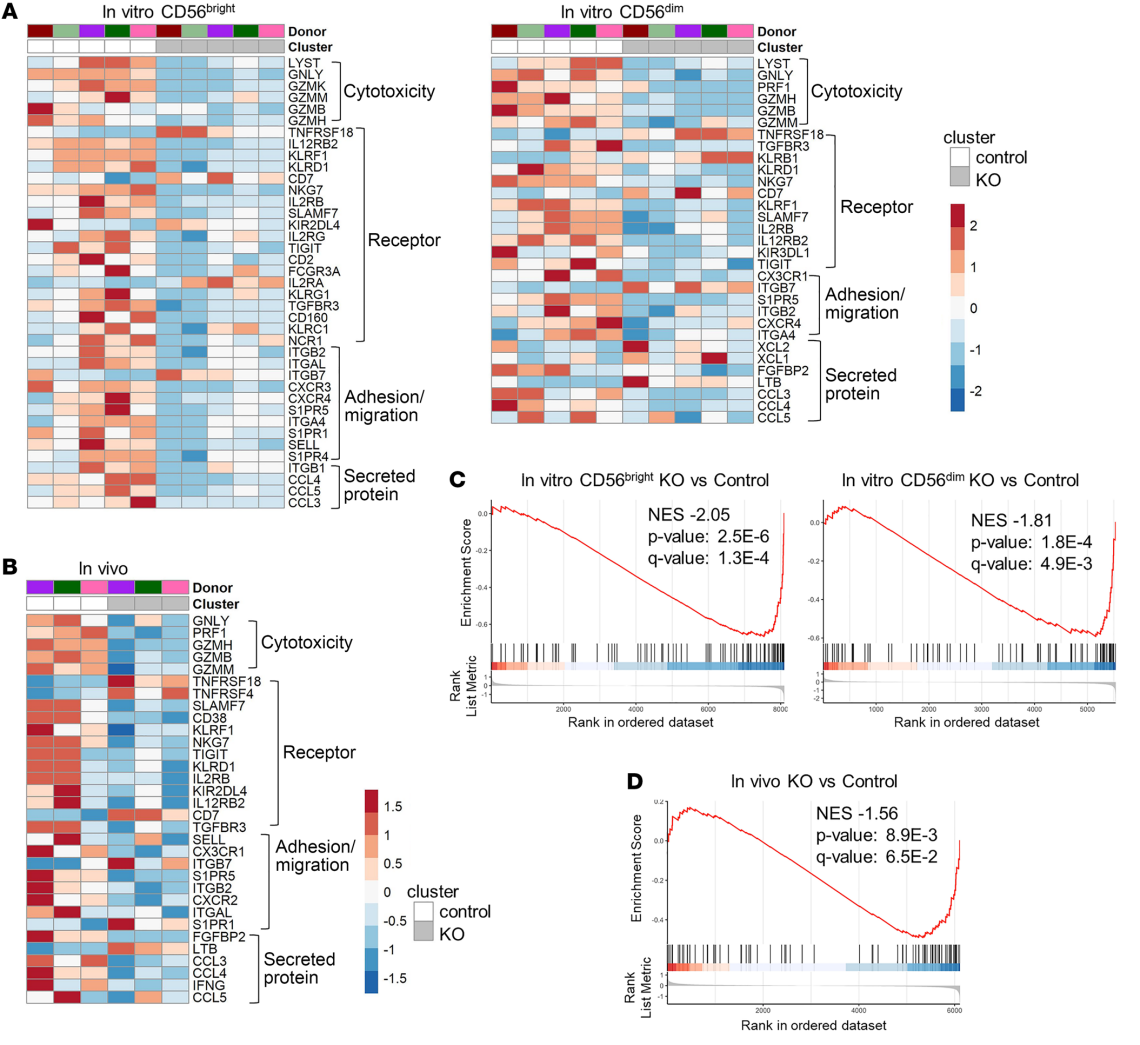
EOMES and T-BET are required to sustain the NK cell transcriptional program. (**A** and **B**) Heatmap of selected DEGs within each subset identified by scRNA-Seq analysis of control and *T+E* edited NK cells in Figure 6. Wilcoxon’s rank-sum test was used for differential analysis with a threshold of adjusted *P* value less than 0.05. (**C** and **D**) Gene set enrichment analysis of the “NK cell medicated cytotoxicity” pathway from the KEGG database in KO versus control clusters.

Differential expression analysis identified numerous genes that were downregulated in KO clusters, many of which were consistently downregulated across the 3 subsets: in vivo, in vitro CD56^bright^, and in vitro CD56^dim^ NK cells (Figures 6 and 7, Supplemental Figure 7, and Supplemental Table 1). Genes responsible for various NK cell functions were altered in KO cells (Figure 6 and Figure 7, A and B). For example, genes that encode several cytotoxic granzymes and chemokines, including CCL5, secreted by NK cells to recruit other cells during an immune response, were also downregulated (33–35). Many NK cell trafficking regulators were downregulated in KO clusters as well, for example *S1PR5*, known to promote NK cell migration (17, 36). The gene expression of the granule protein NKG7, which regulates degranulation in NK cells, was also reduced in KO clusters (37). The KO clusters did have significantly higher expression of some notable genes compared with control clusters, including *TNFRSF18* (GITR), which has been shown to negatively regulate NK proliferation and activation (38–40). Increased expression of integrin *ITGB7* was also observed in all 3 settings. We also observed a decrease in *IL12RB2* transcript expression in KO clusters in in vitro CD56^bright^ and CD56^dim^ NK cells and in vivo (CD56^dim^), which may mechanistically contribute to the decrease of p-STAT4 in response to IL-12 stimulation in NK cells that lack EOMES and T-BET (Figure 7A and Figure 5C).

We performed gene set enrichment analysis (GSEA) to assess Kyoto Encyclopedia of Genes and Genomes (KEGG) pathway enrichment on control versus KO NK cell clusters, and in concordance with the individual genes we observed to be downregulated, the “natural killer cell mediated cytotoxicity” pathway was negatively enriched in KO cells from the in vitro as well as the in vivo samples (Figure 7, C and D). In agreement with the downregulated *PRF1* and *GZMB* transcripts, a significant decrease in their protein expression was observed in DKO NK cells by flow cytometry (Supplemental Figure 8). These data reveal that T-BET and EOMES exert a sustained and ongoing control of key human NK cell effector function mediator genes, and when T-BET and EOMES are deleted, these programs are rapidly curtailed.

### Single knockout of T-BET or EOMES has a modest effect on human NK cell transcriptional profiles.

To investigate the individual contribution of EOMES and T-BET to these changes, control, *TBX21-*edited, *EOMES-*edited, and *T+E* edited NK cells generated in vitro were compared. Control, *TBX21*-edited, and *EOMES*-edited samples had similar UMAP clustering, indicating that single *TBX21* or *EOMES* editing had minimal effect on major NK cell transcriptional profiles (Supplemental Figure 9, A–D). Comparing NK cells with single or double editing of T-box TFs against control samples within CD56^bright^ and CD56^dim^ clusters, *T+E* edited NK cells had more differentially expressed genes (DEGs) than single-edited NK cells in comparison with control (Supplemental Figure 9, E–K). In this analysis, 375 of 527 DEGs identified of all comparisons belonged to the *T+E* edited samples compared against control and were not significant in single-edited sample comparisons — for example, *KLRD1* and *IL2RG* in CD56^bright^ clusters and *PRF1* and *SLAMF7* in CD56^dim^ clusters (Supplemental Figure 9, I–K). Some genes, like *NKG7* (CD56^bright^), *GZMB* (both CD56^bright^ and CD56^dim^), and *S1PR5* (CD56^dim^), were downregulated by single deletion of either *TBX21* or *EOMES*, but the fold change over control was greater when both TFs were edited. *TBX21-*single-edited NK cells had the fewest transcriptional changes (31 and 29 DEGs in CD56^bright^ and CD56^dim^, respectively), while more genes — for example, *TNFRSF18* (CD56^bright^) and *LYST* (CD56^dim^) — were differentially expressed in *EOMES*-edited (113 and 48 DEGs in CD56^bright^ and CD56^dim^, respectively) and *T+E* edited (336 and 304 DEGs in CD56^bright^ and CD56^dim^, respectively) NK cells but not in NK cells that were only *TBX21*-edited (Supplemental Figure 9, I–K). Since the effect of single TBX21 or EOMES CRISPR editing on the transcriptional profile of NK cells was minimal, we focused subsequent analyses on control versus *T+E* edited NK cells.

### T-BET and EOMES regulate expression of other TFs.

Among the significantly differentially expressed genes in the KO versus control clusters of *T+E* edited versus control NK cell samples, we identified TF genes implicated in lymphocyte and NK cell function and identity (Supplemental Figure 9). Expression of *ZEB2*, a known target of T-BET required for mouse NK cell terminal maturation, was significantly decreased in all 3 settings of in vitro CD56^bright^, CD56^dim^, and in vivo KO compared with control clusters (Supplemental Figure 10) (41). RUNX3, critical for innate lymphoid cell (ILC) lineage and function, and BHLHE40 (a cofactor of T-BET), which promotes *IFNG* expression in lymphocytes, were downregulated in the KO clusters of in vitro CD56^dim^ and the in vivo set compared with control clusters (42–46). Two notable TF genes that were altered in KO NK cells in the in vivo setting are *NFATC2* and *KLF2* (Supplemental Figure 10A). NFAT is induced upon activation of NK cells and promotes transcription of *IFNG* in NK cells (47). KLF2, a negative regulator of NK cell proliferation, was increased in KO clusters in vivo (Supplemental Figure 10A) (48). Interestingly *KLF2* expression was lower in the in vitro CD56^bright^ KO cluster compared with control (Supplemental Figure 10C). This demonstrates that the expression and regulatory contributions of TFs downstream of T-BET and EOMES are dependent on both NK subset and context.

In both in vitro CD56^bright^ and CD56^dim^ NK cells, *ETS1*, known for its essential role in NK cell development and in promoting *IFNG* expression, had reduced expression in the KO clusters (Supplemental Figure 10, B and C) (49–51). *BCL11B*, which is a key TF critical for human NK cells to differentiate from CD56^bright^ to CD56^dim^, was also decreased in CD56^dim^ KO clusters (52).

### Loss of T-box TFs results in ILC-3–biased ILC progenitor cells.

**Figure 8:**
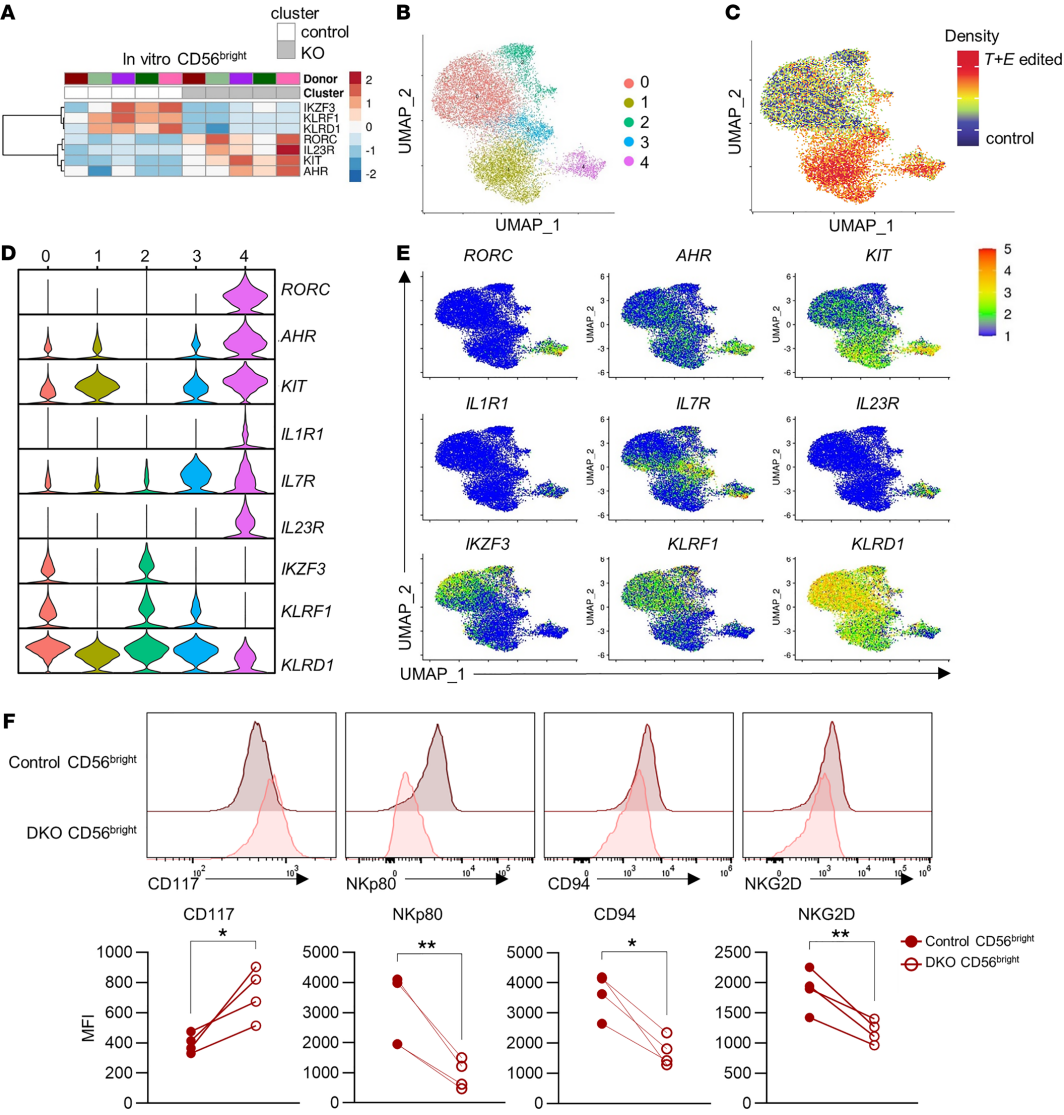
Loss of T-box TFs in NK cells results in ILC-3–biased ILC progenitor cell phenotype. (**A**) Heatmap of differentially expressed ILCP-associated marker genes expressed by in vitro CD56^bright^ KO versus control clusters. (**B**) UMAP of in vitro CD56^bright^ NK cells only. (**C**) UMAP overlaid with density of cells originating from control samples and *T+E* edited samples. (**D**) Violin plot of ILCP-related markers within clusters identified in **B**. (**E**) Expression of indicated ILCP-related markers overlaid on UMAP space. DEGs were determined using Wilcoxon’s rank-sum test and adjusted *P* value of less than 0.05. (**F**) Protein expression of CD117. NKp80, CD94, and NKG2D of in vitro–maintained *TRAC-*edited (control) and gated T-BET/EOMES–DKO cells 8 days after CRISPR electroporation quantified by flow cytometry. *n* = 4 donors, 2 independent experiments. Data were compared with ratio paired *t* test. *P < 0.05, **P < 0.01.

NK cells and ILC-1s are categorized as group 1 ILCs. There are 2 other lineages of innate lymphocytes (ILC-2s and ILC-3s) that produce cytokines analogous to their helper T cell counterparts (53, 54). Accompanying the decreased expression of the T-box TFs that promote NK cell maturation, we observed an increased expression of TFs associated with ILC-3 identity in the KO cluster of in vitro CD56^bright^ NK cells. The expression of the ILC-3–defining TFs *AHR* and *RORC* was significantly higher in the KO compared with the control cluster, consistently across donors (Figure 8A) (54–57). This is in concordance with the increased expression of the immature NK cell/innate lymphocyte markers *KIT* and *IL23R* in these CD56^bright^ KO clusters (Figure 8A) (53, 54). *IKZF3* (AIOLOS) expression, which is normally suppressed in ILC-3s but expressed by NK cells, was also downregulated in CD56^bright^ KO clusters (58, 59). To investigate whether these markers are altered in all versus a subset of cells within the CD56^bright^ KO cluster, clustering and UMAP plotting were performed with only the CD56^bright^ KO and control clusters identified in Figure 6A (Figure 8, B–E). This revealed a cluster (cluster 4), predominantly composed of cells from the *T+E* edited samples, that highly expressed ILC-3–associated markers (Figure 8, D and E). The other cluster predominantly composed of the *T+E* edited NK cells (cluster 1) shared high expression of *KIT* and decreased expression of *KLRD1* and *KLRF1* similar to those in cluster 4. Taking into consideration that the NK cells used in this study were derived from peripheral blood, cluster 1 and cluster 4 (which expressed ILC-3 transcripts) appear most similar to CD117^+^CD56^+^ ILC precursors (ILCPs) that can give rise to both NK cells and ILC-3s, since fully mature ILC-3s are predominantly tissue-resident (60, 61). We validated increased protein expression of CD117, encoded by *KIT*, as well as decreased protein expression of CD94, NKp80, and NKG2D in DKO CD56^bright^ NK cells by flow cytometry, consistent with the expression patterns of these molecules at the ILCP stage of NK development (Figure 8F) (60). These data suggest that T-BET and EOMES are required to actively suppress alternative ILC lineage–defining programs in CD56^bright^ NK cells, and when these T-box TFs are removed, NK cells become ILCP-like and can acquire genes associated with a different ILC lineage.

### T-BET and EOMES regulate chromatin accessibility in human NK cells.

**Figure 9:**
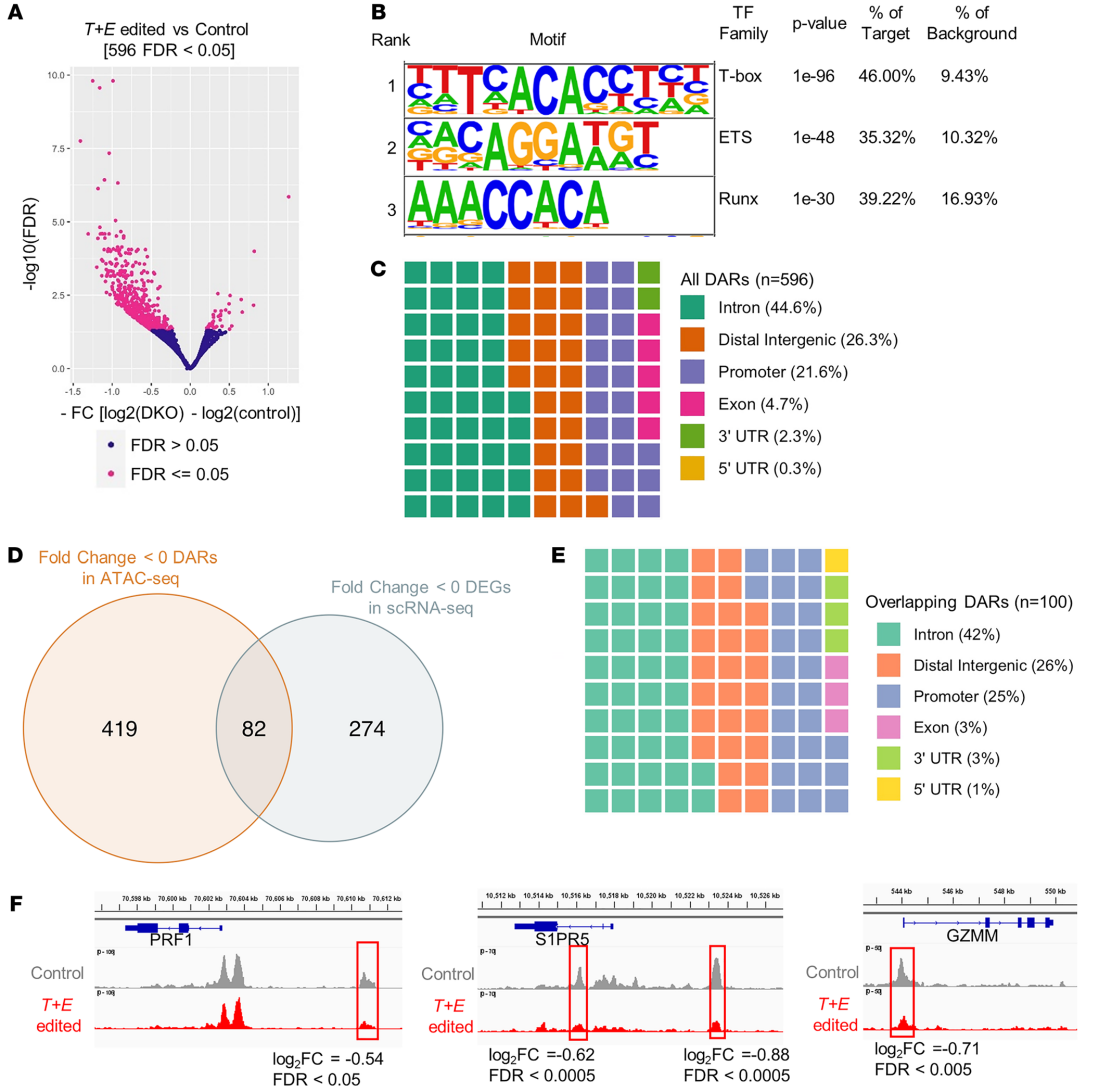
EOMES and T-BET maintain NK cell chromatin accessibility. (**A**) Differentially accessible regions (DARs) identified by DESeq2 (blue, FDR > 0.05; pink, FDR ≤ 0.05). (**B**) HOMER de novo motif enrichment analysis on all DARs using findMotifsGenome.pl with automatically generated background by HOMER. (**C**) Square pie chart of genomic region distribution of all DARs annotated using Chipseeker annotatePeak function. (**D**) Venn diagram of overlapping genes annotated as downregulated DARs in ATAC-seq and genes identified as downregulated DEGs in CD56^bright^ or CD56^dim^ KO clusters in scRNA-Seq. (**E**) Genomic region distribution of DARs (100 regions total, annotated to 82 unique genes) that are annotated to be in or near DEGs. (**F**) Representative peaks in *PRF1*, *S1PR5*, and *GZMM*, regions showing loss of accessibility in *T+E* edited NK cells. *n* = 2 donors, 2 independent experiments.

As T-box TFs have been suggested to act as pioneer factors that can modulate chromatin accessibility, we hypothesized that loss of T-BET and EOMES in mature NK cell subsets consequently results in epigenetic remodeling (16, 62–64). Assay for transposase-accessible chromatin using sequencing (ATAC-seq) was performed on control and *T+E* edited NK cells, to elucidate the impact of T-BET and EOMES deletion on chromatin accessibility. Consistent with our hypothesis, chromatin accessibility was decreased in many genomic regions in the *T+E* edited compared with control NK cells (Figure 9A and Supplemental Table 2). Motif analysis revealed significant enrichment of T-box family motifs within loci that had reduced accessibility in *T+E* edited NK cells. Further, ETS and RUNX motifs were also enriched in these regions, suggesting that T-BET, EOMES, RUNX3, and ETS1 together likely coordinate a large component of the human NK cell molecular program (Figure 9B). Differentially accessible regions (DARs) identified were distributed mainly in introns (44.6%), distal intergenic regions (26.3%), and promoter regions (21.6%) (Figure 9C). Many DARs that were less accessible in *T+E* edited NK cells were in or near DEGs that were decreased in KO clusters (CD56^bright^ and/or CD56^dim^) identified from the scRNA-Seq data (Figure 7 and Figure 9D). The 100 DARs (annotated to 82 unique genes) that overlapped with DEGs were distributed in genomic regions similarly to all the DARs identified (Figure 9E). As examples, decreased peak signals were identified in putative regulatory regions and promoter regions of *PRF1*, *S1PR5*, and *GZMM* whose transcript expression levels were decreased in KO clusters of the scRNA-Seq experiment (Figure 7, Figure 9F, and Supplemental Figure 7). This suggests that T-BET and EOMES maintain accessibility of critical regulatory regions of genes, thereby sustaining the NK cell transcriptional program.

## Discussion

In this paper, we report that ongoing transcriptional regulation by T-BET and EOMES is required for proper function of mature human NK cells. Deletion of both EOMES and T-BET resulted in reduced ability of human NK cells to control tumor targets in vivo. Mechanistically, this is explained by reduced proliferation, impaired cytokine-receptor signaling, and defective NK cell effector functions. Cytokine production was reduced when NK cells lacked one or both TFs. T-BET– and EOMES-DKO cells produced almost no IFN-γ and had compromised IL-15 and IL-12 receptor signaling. NK cells lacking both T-BET and EOMES also had reduced degranulation to tumor targets after a more prolonged time period following the CRISPR deletion. Further, T-BET and EOMES single deletion had differential effects on the function of CD56^bright^ and CD56^dim^ NK cell subsets, suggesting both redundant and unique regulatory roles in an NK cell subset–specific fashion. Finally, by scRNA-Seq analysis, NK cells demonstrated a profound loss of the NK cell functional and identity-defining transcriptional programs, as well as the unexpected emergence of an ILC-3–biased ILCP molecular program. Collectively, these findings demonstrate that EOMES and T-BET are required for fully developed NK cells to properly respond to stimuli, as well as to maintain NK cell identity.

The role of EOMES and T-BET in initiation of NK cell development is well established in mouse models. Mice with global knockout of T-BET lack mature NK cells in the periphery (15, 16). Similarly, in hematopoietic compartment–specific and constitutive NK cell–specific EOMES-KO mouse models, mature NK cell numbers are markedly reduced (15, 65). Likewise, the importance of T-BET in human NK cell development was evidenced by a patient with T-BET deficiency, who had an impaired group 1 ILC compartment (20). Forced overexpression of T-BET and EOMES can accelerate in vitro NK cell differentiation from cord blood, but these studies do not inform their requirement for maintenance of mature NK cell function or molecular program (23). Collectively, these published studies have defined the clear requirement for T-box TFs to *initiate* NK cell molecular programs, as they develop from multipotential progenitors. However, these models are generally not sufficient to understand the ongoing importance of, and genetic programs sustained by, T-box TFs, which dynamically alter expression levels during NK cell maturation (27).

Recently, our group developed an inducible NK cell-specific EOMES KO mouse model that revealed the requirement of sustained EOMES expression for proper murine NK cell effector functions and the homeostasis of stage II and III murine NK cells (18). This contrasts with the EOMES-deleted human NK cells reported here, where human NK cell subset–specific functions are minimally affected, without impacting NK cell survival. Based on these observations, the study of T-box TFs within human NK cells is required to understand their role in human NK cell molecular programs. Here, we show that single deletion had modest impact on human NK cell functions and transcriptional profile compared with double T-BET and EOMES deletion. These findings contrast with mouse studies showing functional defects and widespread transcriptional changes in T-BET– or EOMES-single-KO NK cells. This again highlights distinctions between murine and human NK cell transcriptional control (18, 66). Moreover, there have been no murine studies of simultaneous T-BET and EOMES conditional and inducible deletion to date. Our data demonstrate that T-BET and EOMES exhibit redundancy in human NK cells in terms of functional regulation as well as transcriptional regulation. Thus, our finding that EOMES and T-BET are of profound and critical importance for ongoing human NK cell functions and identity provides important new insights into the NK cell molecular program, including key downstream TFs impacted by EOMES and T-BET regulation.

scRNA-Seq analysis of CRISPR-edited primary human NK cells revealed EOMES and T-BET regulated key NK cell functional pathway genes, including cytotoxic effector molecules, NK cell receptors, and trafficking and migration regulators (e.g., chemokines and chemokine receptors). Using a loss-of-function approach, several TFs were discovered to be directly regulated by T-BET and EOMES, many of which have roles in regulating *IFNG* transcription in other cell types. For example, in both CD56^bright^ and CD56^dim^ T-BET– and EOMES-KO human NK cells, a reduced expression of ZEB2 was observed, which is a direct target of T-BET in mouse NK cells and is required for mouse NK cells to mature and acquire optimal function (41). Moreover, T-BET– and EOMES-KO NK cells have reduction of RUNX3, which has been shown to be critical for ILC lineage initiation, cytotoxic molecule expression, and IL-15–induced proliferation (42–44). Further, BHLHE40, a cofactor of T-BET that normally promotes *IFNG* expression in lymphocytes, also had markedly decreased expression in DKO human NK cells (45, 46). The downregulation of ETS1 in DKO NK clusters likely contributed to the reduction of IFN-γ upon IL-12 stimulation in *T+E* edited NK cells (49–51). Collectively, the approach of T-box TF deletion followed by analysis of RNA expression in single cells reveals critical links between T-BET, EOMES, and these transcriptional regulators, providing evidence of their ongoing regulation by T-box TFs in human NK cells.

In the more immature CD56^bright^ NK cell compartment, we observed a reversion of NK cells to have ILCP-like marker expression pattern and an ILC-3–biased ILCP population that was specifically enriched in the T-BET– and EOMES-KO samples. This cluster resembles the previously described CD56^+^ ILCPs in its high expression of CD117 and IL23R (60). Consistent with the description that the CD56^+^ ILCPs can give rise to group 1 and group 3 ILCs, this ILCP-like cluster observed in our study seems to be biased toward the ILC-3 lineage with its high expression of the ILC-3–associated TFs RORC and AHR and low expression of IKZF3 (58, 61). This observation is also consistent with functional plasticity between ILC groups, in this case mature human NK cells and ILCPs, governed by expression levels of ILC group–specific TFs (58, 65, 67). Our data suggest that EOMES and T-BET suppress non-NK ILC lineages, with a bias specifically against the ILC-3 lineage, and when EOMES and T-BET are deleted, NK cells acquire a transcriptional signature that matches that of an ILC-3–biased ILCP.

This study also revealed that T-BET and EOMES not only regulate transcript expression in NK cells, but also participate in maintaining chromatin accessibility. Abrogation of T-BET and EOMES led to decreased chromatin accessibility near the *PRF1* and *S1PR5* loci, both of which had decreased transcript expression in *T+E* edited NK cells, among many other NK cell effector function–related gene loci that were also transcriptionally affected. The reduced chromatin accessibility upon T-box TF deletion indicates an active role in maintaining the NK cell state, and reveals a new layer of regulation by T-BET and EOMES beyond transcriptional regulation, as they are critical for NK cell chromatin states.

NK cellular therapy is a promising cancer immunotherapy, since tumor cells commonly express NK-activating ligands as well as downregulate MHC class I molecules, which render them sensitive to NK cell–mediated clearance (3, 4). Expression of T-BET and EOMES is negatively regulated by immunosuppressive cytokines like TGF-β in tumors (68, 69). In a mouse adoptive transfer model, reduced EOMES and T-BET expression over time in NK cells after adoptive transfer correlated with reduced IFN-γ production and impaired long-term tumor control (70). Our loss-of-function study showed that T-BET and EOMES in mature NK cells are indeed required for NK cell function, and thus reduction of T-box TFs directly limits NK cell functional capacity. As our study focused on conventional, peripheral blood NK cells, future work should investigate the expression and importance of T-BET and EOMES in various NK therapeutic approaches such as cord blood–derived NK cells, induced pluripotent stem cell–derived NK cells, cytokine-induced memory-like NK cells, and NK cells transduced with chimeric antigen receptors (5, 12, 71–74). The expression levels and kinetics of T-BET and EOMES in those settings could be potentially utilized as a measure of NK cell identity integrity and optimal function.

In summary, this study reveals that EOMES and T-BET are required for sustaining mature NK cell identity and functional activity. The deletion of EOMES and T-BET led to functional, proliferative, and signaling defects that resulted in an impaired response against tumor cells in vivo. This illustrates the importance of maintaining T-BET and EOMES expression for optimal NK cell antitumor responses. Moreover, deletion of these NK cell identity TFs results in emergence of an ILC-3–biased ILCP program that may represent a default developmental pathway. Future studies that interrogate the role of genes that are directly regulated by T-BET and EOMES revealed in our study, and that are indirectly regulated through other TFs regulated by T-BET and EOMES, will be important steps to further elucidate how NK cells orchestrate a transcription network responsible for mature NK cell responses.

## Methods

### Human NK cell isolation and culture.

Healthy donor NK cells were isolated from leukapheresis chamber from platelet donors using RosetteSep (STEMCELL Technologies) (routinely >95% CD56^+^CD3^–^) followed by Ficoll-Paque PLUS (GE Healthcare) centrifugation. NK cells were maintained in low-dose (1–3 ng/mL) IL-15 in RPMI 1640 plus 10% heat-inactivated human AB serum plus 10 mM HEPES plus 1× penicillin/streptomycin plus 1% of non-essential amino acids, sodium pyruvate, and l-glutamine as previously described (9), with media changes every other day.

### Mice.

NOD-*scid* IL2Rg^null^ (NSG) mice were purchased from The Jackson Laboratory (RRID:IMSR_JAX:005557). Mice were then bred and maintained in specific pathogen–free housing, and experiments were conducted in accordance with the guidelines of and with the approval of the Washington University Animal Studies Committee. Experiments were performed on 7- to 16-week-old male and female mice. Within each experiment, mice were age and sex matched.

### Cell lines.

K562 cells were obtained from ATCC and authenticated in 2015 by SNP analysis. K562 cells were cultured in RPMI 1640 medium plus 10% heat-inactivated FBS plus 10 mM HEPES plus 1× penicillin/streptomycin plus 1% of non-essential amino acids, sodium pyruvate, and l-glutamine.

### CRISPR editing of human NK cells.

Freshly isolated NK cells were rested in low-dose IL-15 (1–3 ng/mL) overnight. The next day, NK cells were harvested, washed with PBS, and resuspended in MaxCyte EP Buffer at concentrations recommended by the manufacturer. Cas9 mRNA and gRNA were introduced into the NK cells by electroporation using the protocol WUSTL-2 on the MaxCyte GT electroporation machine. Cells were incubated for 10 minutes at 37°C immediately after electroporation. Low-dose IL-15 medium was added after the incubation. Media changes were performed every 2–3 days. Synthetic sgRNAs were produced by Synthego with modifications (2′-*O*-methyl at first 3 and last bases and 3′ phosphorothioate bonds between first 3 and last 2 bases). The sgRNA sequences were as follows: *EOMES*, AACCAGTATTAGGAGACTCT; *TBX21*, CACCACTGGCGGTACCAGAG; *TRAC*, GAGAATCAAAATCGGTGAAT.

### Apoptosis assessment.

On day 6/7 after CRISPR electroporation, NK cells were harvested and assessed for apoptosis using annexin V and 7-aminoactinomycin D (Sigma-Aldrich) in 1× Annexin V Binding Buffer (Thermo Fisher Scientific) after surface marker staining for flow cytometry analysis.

### NSG xenograft and tumor model.

The next day after CRISPR electroporation, 1 × 10^6^ NK cells were washed with PBS and injected into NSG mice i.v.; the cells were then supported with 1 μg rhIL-15 i.p. 3 times per week. For K562 tumor challenge experiments, approximately 1.5 × 10^6^ luciferase-expressing K562 cells were injected i.v. 4 days after NK cell injection, and 100 ng/mouse rhIL-15 was used to support NK cells for the duration of the experiment after tumor injection. Bioluminescent imaging (BLI) was performed twice a week on an AMI imager (Spectral Instruments Imaging) 10 minutes after i.p. injection of 150 mg/kg d-luciferin. Quantification of BLI signals was performed using Aura software (Spectral Instruments Imaging).

For proliferation assessment, NK cells were washed with PBS and incubated with 1:2,000 CellTrace Violet (Invitrogen) following the manufacturer’s protocol for labeling before injection into the mice. Dye dilution was tracked at time of mouse harvesting by flow cytometry.

For experiments assessing persistence, proliferation, and ex vivo functionality, NK cells were maintained with 1 μg/mouse rhIL-15 i.p. 3 times per week for the entire course of the study.

### Flow cytometry.

Surface marker staining was performed in PBS plus 1 mM EDTA plus 2% heat-inactivated FBS at 4°C in the presence of heat-inactivated goat serum (Sigma-Aldrich). Mouse F_c_‑Block (BD Biosciences) was also used in NSG mice experiments. Intracellular staining was performed with the eBioscience FoxP3 staining kit following the manufacturer’s protocol. Antibodies used are listed in Supplemental Methods. Data were acquired on Beckman Coulter Gallios and Thermo Fisher Scientific Attune flow cytometers and analyzed using FlowJo (Tree Star).

### Phospho-signaling assessment.

On day 6/7 after CRISPR electroporation, NK cells were harvested from tissue culture plates, and cytokine-containing medium was washed off and replaced with cytokine-free medium. NK cells were rested in cytokine-free medium for 30 minutes up to 2 hours. In each experiment an equal number of NK cells, up to 200,000 NK cells, were plated for each condition. Then IL-15 and IL-12 were used to stimulate NK cells (timing and concentration are indicated in figure legends). At the end of incubation, NK cells were fixed with prewarmed 1% paraformaldehyde and permeabilized using ice-cold methanol. Cells were washed 3 times with FACS buffer before staining with surface markers and phospho-antibodies overnight at 4°C.

### Assessment of degranulation and cytokine production.

For in vitro–maintained cells, unless otherwise indicated, on day 6/7 after electroporation up to 200,000 NK cells were plated in 1 well of a 96-well U-bottom plate for each condition. In each independent experiment, an equal number of NK cells were plated for all samples. Stimulation conditions were as follows: K562 at the ratio of 5 NK to 1 K562; 5 ng/mL IL-12 + 25 ng/mL IL-15; 5 ng/mL IL-12 + 25 ng/mL IL-15 + 5 ng/mL IL-18. Immediately after stimulation began, anti-CD107a antibody was added to all wells of the assay. After 1 hour, GolgiPlug and GolgiStop (BD Biosciences) were added and the assay was incubated for 5 hours more, for a total of 6 hours. Then flow cytometry staining was performed as described above to assess intracellular IFN-γ and TNF.

Ex vivo assessment was performed similarly but with isolated splenocytes 1.5–2 weeks after injection of NK cells into NSG mice. Stimulation conditions were as follows: K562 at the ratio of 10 splenocytes to 1 K562; 20 ng/mL IL-12 + 100 ng/mL IL-15; 20 ng/mL IL-12 + 100 ng/mL IL-15 + 20 ng/mL IL-18.

For PMA/ionomycin stimulation experiments, NK cells were stimulated with 1× eBioscience Cell Stimulation Cocktail. After 2 hours, GolgiPlug and GolgiStop were added and the assay was incubated for 4 hours more, for a total of 4 hours. Then flow cytometry staining was performed as described above to assess intracellular IFN-γ.

### Single-cell RNA-Seq.

For each donor, live human NK cells (Zombie^–^mCD45^–^hCD45^+^hCD3^–^CD56^+^) were sorted from splenocytes of NSG mice 7 days after injection using the BD FACSAria Cell Sorter (>98% purity). In parallel, NK cells that were injected into NSG mice were also cultured in vitro in 1 ng/mL IL-15, harvested, and subjected to flow sorting at the same time as the in vivo–maintained human NK cells followed by 10x Genomics scRNA-Seq (5′ v2 chemistry). The resulting data were analyzed as previously described using CellRanger (v6.0, 10x Genomics) with genome alignment to GRCh38; downstream analysis was performed using Seurat v4 (10, 11). Detailed quality control and filtering steps are described in Supplemental Methods.

Resulting clusters were first assigned to be CD56^bright^ or CD56^dim^ and cycling clusters, where cells have high expression of S phase– and G_2_M phase–associated genes. Then clusters were grouped into the following: “KO,” non-cycling clusters where more than 75% of cells within the cluster originate from *T+E* edited samples; and “control,” non-cycling clusters where fewer than 75% of cells are *T+E* edited samples. “Control” and “KO” clusters were then reclustered for visualization. Differential gene expression analysis was performed using Wilcoxon’s rank-sum test implemented in the Seurat FindMarkers function (parameters: logfc.threshold = 0.25, min.pct = 0.1). Gene set enrichment analysis was performed using the R package clusterProfiler (75).

### Assay for transposase-accessible chromatin using sequencing.

CRISPR-edited NK cells were harvested from tissue culture plates on day 7 and day 10 after CRISPR electroporation. Nuclei and libraries were prepared for assay for transposase-accessible chromatin using sequencing (ATAC-seq) following established protocol using Illumina kits (76). The samples from the 2 time points were treated as technical replicates in the analysis using standard ATAC-seq analysis pipeline and tools. Briefly, paired-end reads were trimmed for adaptors and low-quality reads were removed using Cutadapt (v3.2) (77). Trimmed reads were aligned to the *Homo sapiens* genome assembly hg38 using Bowtie2 (v2.4.1) (78, 79). Samtools (v1.3.1) (80) was used to filter reads by alignment score and remove mitochondrial reads, and PCR duplicates were removed using Picard (v2.25.0) (https://broadinstitute.github.io/picard/). Peak calling was performed using Genrich (v0.6) (https://github.com/jsh58/Genrich) ATAC-seq mode (-j -d 100). Differential accessibility analysis was determined by DESeq2 using DiffBind (v4.2) and annotated using Chipseeker (v1.28.3) R packages. De novo motif enrichment analysis on all differentially accessible genomic regions was performed with HOMER (v4.11) using findMotifsGenome.pl with automatically generated background by HOMER.

### Data availability.

The scRNA-Seq (GEO GSE227636) and ATAC-seq (GEO GSE227878) data were deposited to the NCBI’s Gene Expression Omnibus database (GEO). No new code was generated in this study; all analyses were performed using existing packages. Specific parameters used to analyze the data are indicated in Methods and Supplemental Methods.

### Statistics.

Statistical comparisons were performed as indicated in each figure using GraphPad Prism (v9) software or in R. Data are represented as mean ± SEM, and all significance testing comparisons are 2-sided. The specific statistical tests and the sample size are indicated in the respective figure legends. *P* < 0.05 was considered statistically significant.

### Study approval.

All animal studies were approved by the Washington University IACUC and experiments were conducted in accordance with the guidelines of and with approval by the Washington University Animal Studies Committee.

## Author contributions

PW and TAF conceptualized the project. PW, JAF, MMBE, and TAF developed the methodology. PW, JAF, LC, CCN, TY, CCC, JT, SKS, SP, NJ, DARG, NDM, M Gang, JAW, AYZ, MTJ, MF, TS, LM, EM, PP, MBH, BF, AAP, OLG, M Griffith, and MMBE performed the investigation and formal analysis. PW and TAF wrote the original draft of the manuscript. All authors wrote, reviewed, and edited the manuscript.

## Supplementary Material

Supplemental data

Supplemental table 1

Supplemental table 2

Supporting data values

## Version 1 06/06/2023

In-Press Preview

## Version 2 07/03/2023

Electronic publication
