# Supplementary material for: T-BET and EOMES sustain mature human NK cell identity and antitumor function
Source: J Clin Invest. 2023 Jul 3;133(13):e162530. doi: 10.1172/JCI162530 (PMC10313375; doi:10.1172/JCI162530)
Supplement: Supplemental data [file jci-133-162530-s115.pdf]

SUPPLEMENTAL METHODS

Flow Cytometry Antibodies

The following antibody clones and reagents from indicated manufacturers were used:

Beckman Coulter: CD56 (N901), CD3 (UCHT1), CD45 (J33); Life Technologies: CD16 (3G8); Biolegend: T-BET (4B10), IFN-γ (4S.B3), PERFORIN (dG9), TNF (Mab11), CD107a (H4A3), GZMB (GB12), CD117 (104D2), CD94(DX22), CD122 (TU27); eBioscience: EOMES (WD-1928); BD: Annexin V, AKTpS473 (M89-61), STAT4pY693 (38/p-Stat4), ERK1/2 pT202/pY204 (20A), mouse CD45 (30-F11), mouse FcBlock; R&D systems: NKp80 (239127); Invitrogen: NKG2D (1D11).

Western Blotting

CRISPR-edited NK cells 1 week after electroporation were spundown and resuspended in 1X RIPA Buffer (CST) supplemented with 1X Protease Inhibitor Cocktail (CST). Lysates were ran on NuPAGE™ 4 to 12% Bis-Tris Mini Protein Gel (Thermo Fisher) along with Precision Plus Protein™ WesternC™ Blotting Standards *(Biorad), and transferred using the iBlot2 Western Blot Transfer System to nitrocellulose membrane(Thermo Fisher). Membrane was blocked in 5% Nonfat dry milk (Biorad) prior to blocking with primary antibody. The following antibodies purchased from Cell Signaling Technology were used: STAT44 (C46B10) Rabbit mAb, AKT (pan) (C67E7) Rabbit mAB, p44/42 MAPK (Erk1/2) (137F5) Rabbit mAb, β-Actin (8H10D10) Mouse mAb, Anti-rabbit IgG HRP-linked Antibody, Anti-mouse IgG HRP-linked Antibody. Blots were incubated in SuperSignal West Pico Plus ECL substrate (34577) prior to imaging on the Chemidoc MP system(Biorad). When necessary, Restore™ PLUS Western Blot Stripping

Buffer (Thermo Fisher) was used following manufacturer's protocol. Quantification of bands were performed using ImageJ.

In vitro Flow-Based killing Assay

CRIPSR-edited NK cells were sorted into CD56^{bright} (CD56^{high} CD16-) and CD56^{low}(CD56^{low} CD16+) 5 days after CRISPR and rested in 3 ng/mL IL-15. The next day, NK cells were incubated with CFSE-labeled K562 target cells for 4-6 hours in the presence of 3ng/mL IL-15. 7-AAD staining was performed at the end of the incubation and assessed by flow cytometry to determine % specific lysis, as previously described(1).

Incucyte Killing Assay

GFP-expressing SKOV-3 cells (ATCC) were cultured in McCoy 5A+ 10% FBS + 1% Pen/Strep and plated at 10,000 cells/well at least 1 hour prior to addition of NK cells. CRISPR-edited NK cells were incubated with the plated target cells at indicated effector to target ratios in the presence of 1ng/mL IL-15 for 6 days. SKOV-3 numbers (Green object count) were monitored over the course of 6 days on the Incucyte Live-Cell Analysis system (Sartorius).

Single-Cell RNA-Sequencing Analysis

For each of the 2 donor samples in Fig. S7, the 4 experimental conditions were stained with hashtag oligonucleotide-tagged (HTO) antibodies, pooled together, and ran as one run for CITE-seq andfastq files were aligned as above with the addition of a feature barcoding reference file for hashtagging alignment and count as previously described(2). Experimental conditions were demultiplexed in Seurat (v4) by first removing dead cells (percent mitochondrial < 10%), and cells

with extremely high or low gene counts (nFeature_RNA > 500; nCount_RNA < 25000 (Dnr1) or nCount_RNA < 40000 (Dnr2); nCount_HTO < 8000), followed by normalizing antibody reads using centered log ratio by feature followed by HTODemux (quantile = 0.99) retaining all cells positively assigned to one antibody (Hashtag-2: dTRAC; Hashtag-3: dTbet; Hashtag-4: dEomes; Hashtag-5: DKO). The 2 donors' control and T+E edited samples were then pooled with additional 3 donor in vitro samples for analyses in Figure 6-8. For the 3 additional donors used in scRNA-seq in Figures 6-7, Low quality and dead cells were filtered using the following thresholds: RNA features > 200 & percent mitochondrial reads < 10 to 12.5 (sample dependent). Significant principle components were chosen by JackStraw Analysis and Elbow Plot followed by louvain unsupervised clustering and UMAP (3). Batch correction was performed grouped by donor using Harmony R package (4). Transcription factor genes presented were only those included in the "Collection of known and likely human TFs" by Lambert et al(5).

Supplementary References

1. Berrien-Elliott MM, et al. Multidimensional Analyses of Donor Memory-Like NK Cells Reveal New Associations with Response after Adoptive Immunotherapy for Leukemia. *Cancer Discov.* 2020;10(12):1854–1871.
2. Berrien-Elliott MM, et al. Hematopoietic Cell Transplantation Donor-derived Memory-Like NK Cells Functionally Persist after Transfer into Patients with Leukemia. *Sci. Transl. Med.* 2022;14(633):eabm1375.
3. Hao Y, et al. Integrated analysis of multimodal single-cell data. *Cell* 2021;184(13):3573-3587.e29.
4. Korsunsky I, et al. Fast, sensitive and accurate integration of single-cell data with

Harmony. *Nat. Methods 2019 1612* 2019;16(12):1289–1296.

5. Lambert SA, et al. The Human Transcription Factors. *Cell* 2018;172(4):650–665.

Supplemental Figure 1

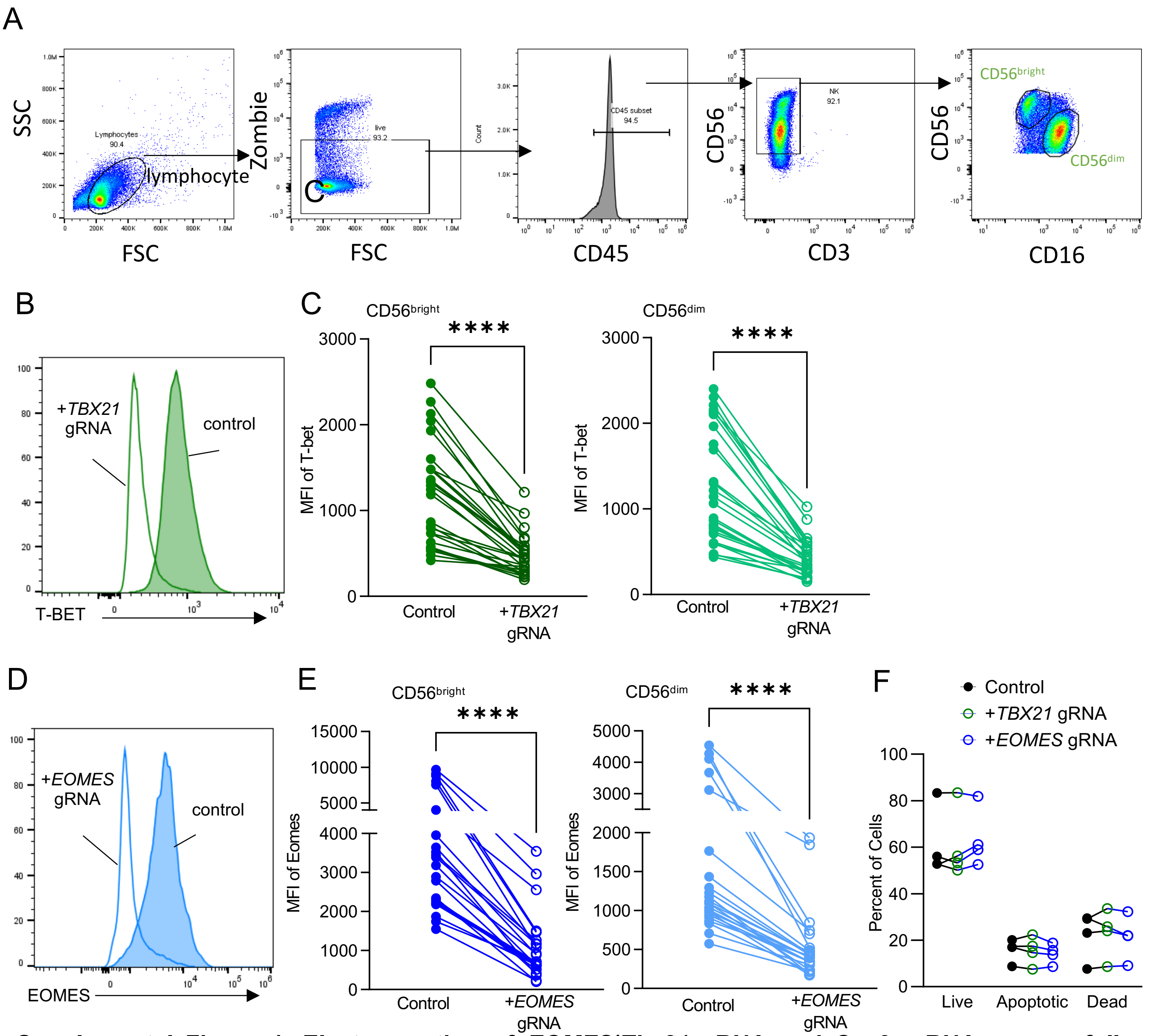

Supplemental Figure 1. Electroporation of *EOMES/Tbx21* gRNA and Cas9 mRNA successfully abrogates EOMES and T-BET protein expression in unexpanded primary human NK cells. (A) Example flow gating of human NK cells maintained in vitro. (B-E) NK cells were subjected to electroporation to deliver Cas9 mRNA with respective sgRNA using the Maxcyte GT system. NK cells were cultured in low dose IL-15, and on D6/7 T-BET and EOMES protein expression was assessed by flow cytometry. (B) Representative flow histogram plot and (C) summary data of T-BET protein expression in NK cells electroporated with Cas9 mRNA and gRNA targeting *TBX21* locus in CD56^{bright} and CD56^{dim} NK cells identify by flow gating. (D) Representative histogram plot and (E) summary data of EOMES protein expression in NK cells electroporated with Cas9 mRNA and gRNA targeting *EOMES* genomic locus in CD56^{bright} and CD56^{dim} NK cells gated by flow. (B-E) n=23-27 healthy donors, 18-24 independent experiments. (F) NK cells were cultured in low dose IL-15 then harvested on Day 6/7, stained for Annexin V and 7-AAD, and analyzed by flow cytometry. n=4 donors, 4 independent experiments. (C,E) Data were compared using ratio paired t-tests. (F) Data were compared with 2 way ANOVA with Holm-Šídák multiple comparison test.

Supplemental Figure 2

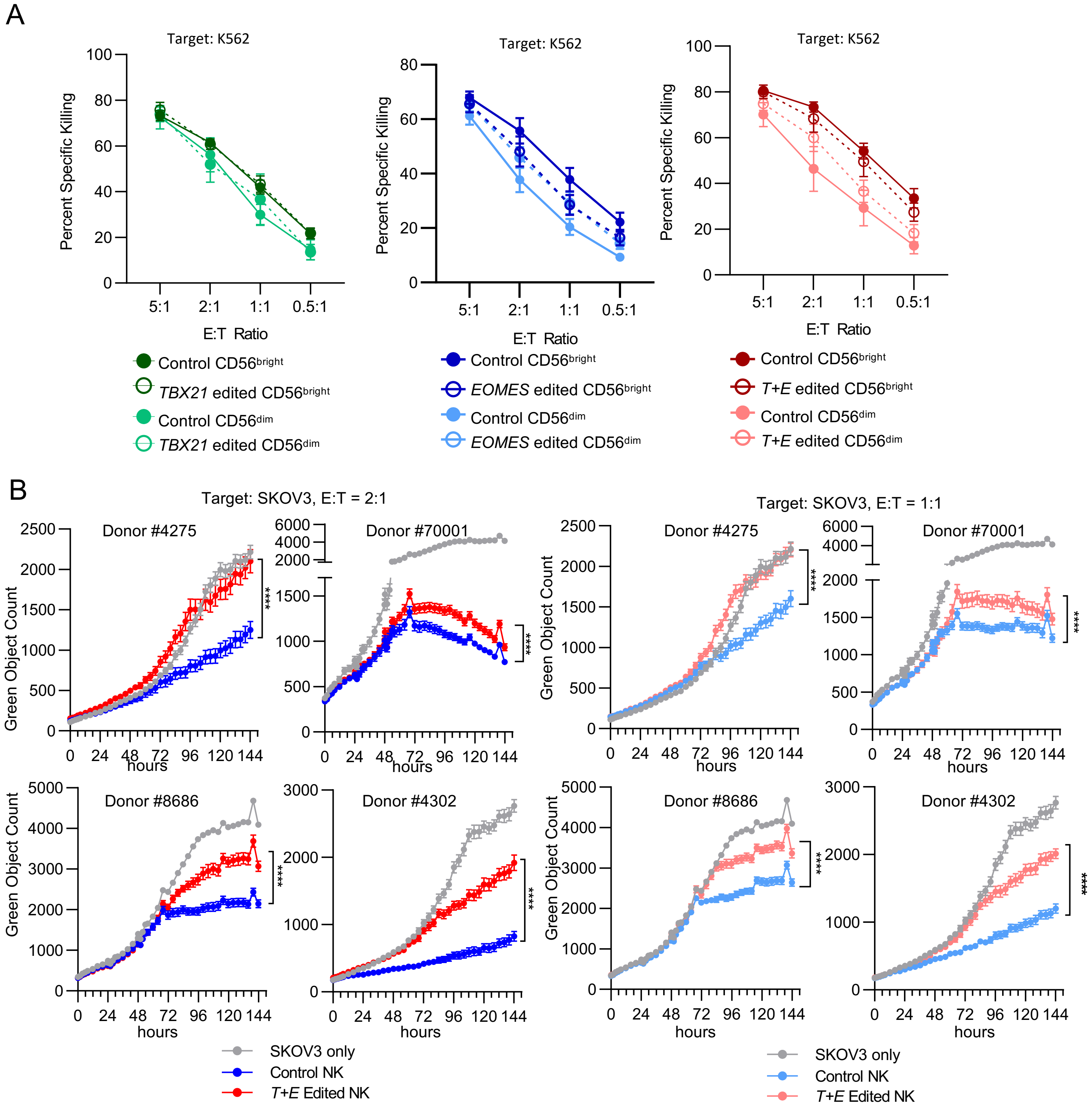

Supplemental Figure 2. EOMES and T-BET are not required for short-term killing of target cells but are required for long-term killing. (A) Sorted CD56bright and CD56dim primary human NK cells were incubated with CFSE-labeled K562 targets at the indicated effector:target (E:T) ratios 5 days after electroporation with Cas9 mRNA. 4-6 hours after, cells were harvested and stained with 7-AAD to determine percent specific killing of CFSE+ targets by NK cells. (B) Incucyte long-term killing against the ovarian cancer cell line SKOV3. Six days after electroporation, *T+E* edited or control NK cells were co-cultured with GFP-expressing SKOV3 at the indicated E:T ratio. Green object counts(SKOV3) were monitored over 6 days. Data were compared using 2-way ANOVA.

Supplemental Figure 3

A

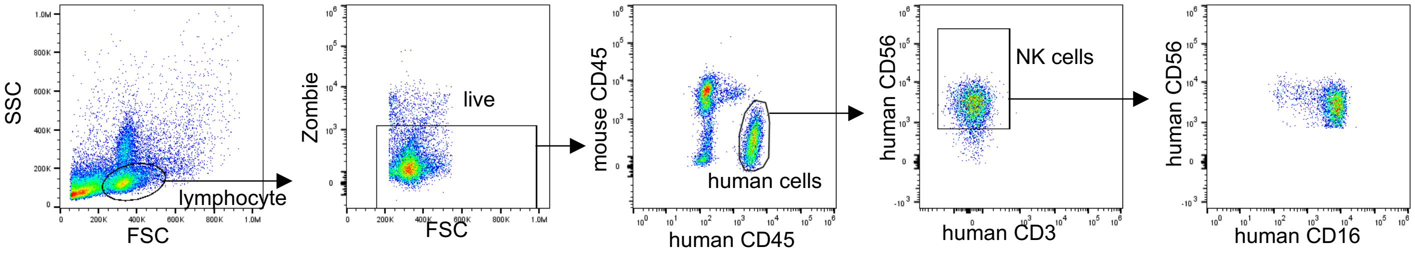

B

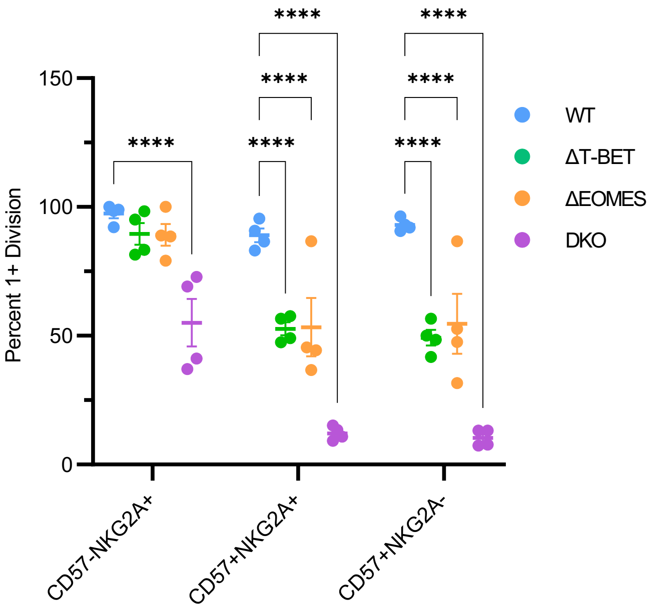

Supplemental Figure 3. EOMES and T-BET are required for NK cell persistence and proliferation in vivo especially in less mature subsets of NK cells. (A) Example flow gating of human NK cells maintained in vivo in NSG mice. (B) *T+E* edited NK cells were labeled with CellTrace Violet(CTV) dye prior to injection into NSG mice. At 1.5-2 weeks after injection, NK cells were harvested and CTV dilution was analyzed by flow cytometry. Summary data of percentage of NK cells that have divided at least once. N=4 mice from 3 independent experiments. Data were compared using 2-way ANOVA with Holm-Šídák multiple comparison test.

Supplemental Figure 4

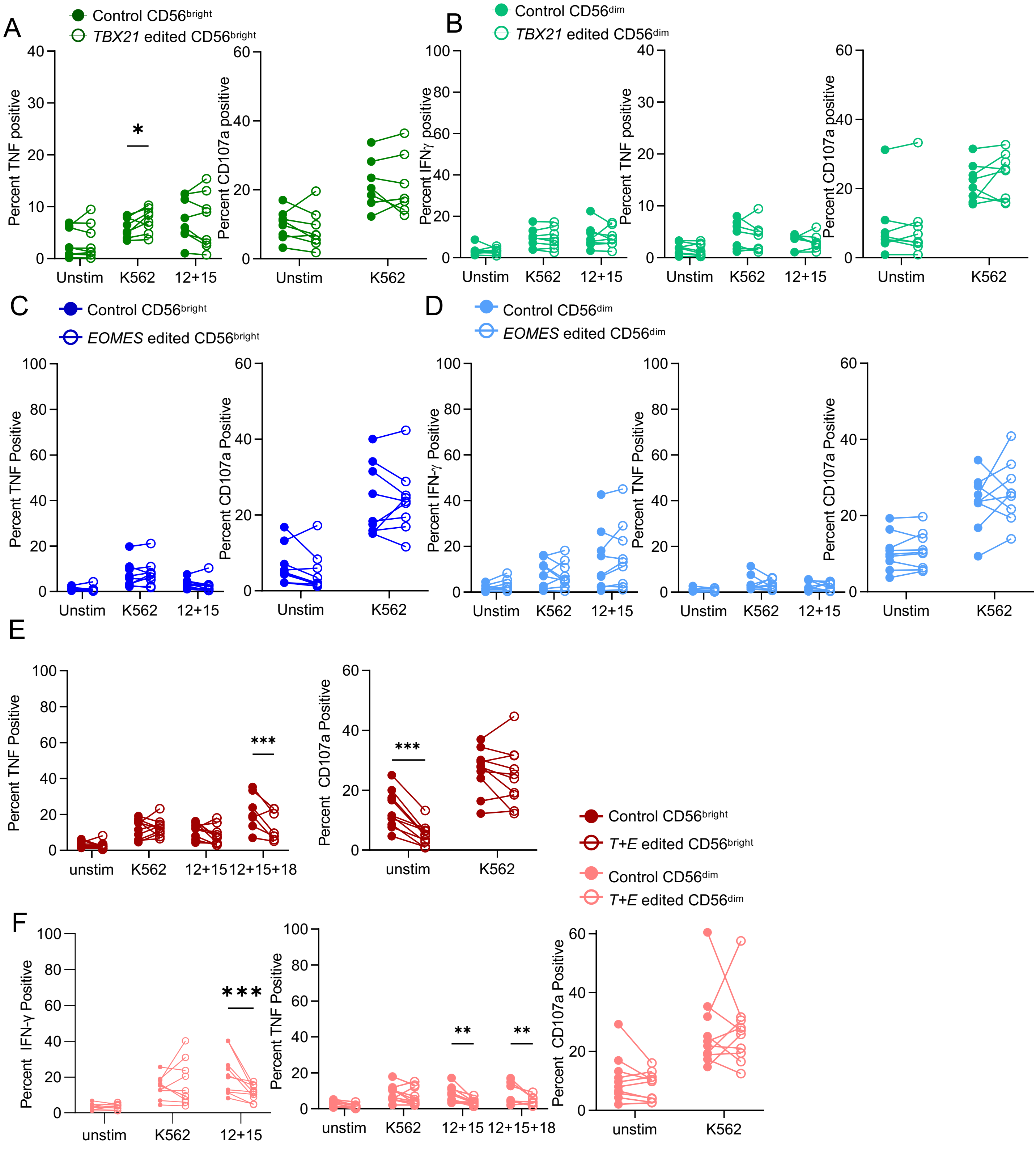

Supplemental Figure 4. **NK cell in vitro effector functions are profoundly impaired in *T+E* edited NK cells.** In vitro functional assessment on Day 6/7 after CRISPR-editing, NK cells were stimulated with K562 and IL12+15. Degranulation (CD107a) and cytokine production were quantified by intracellular flow staining in (A-B) *TBX21* edited, (C-D) *EOMES* edited, and (E-F) *T+E* edited NK cells flow gated to be CD56^{bright} or CD56^{dim}. n=7-10 donors, 4-7 independent experiments. Data were compared by 2-way ANOVA with Holm-Šídák multiple comparison test.

Supplemental Figure 5

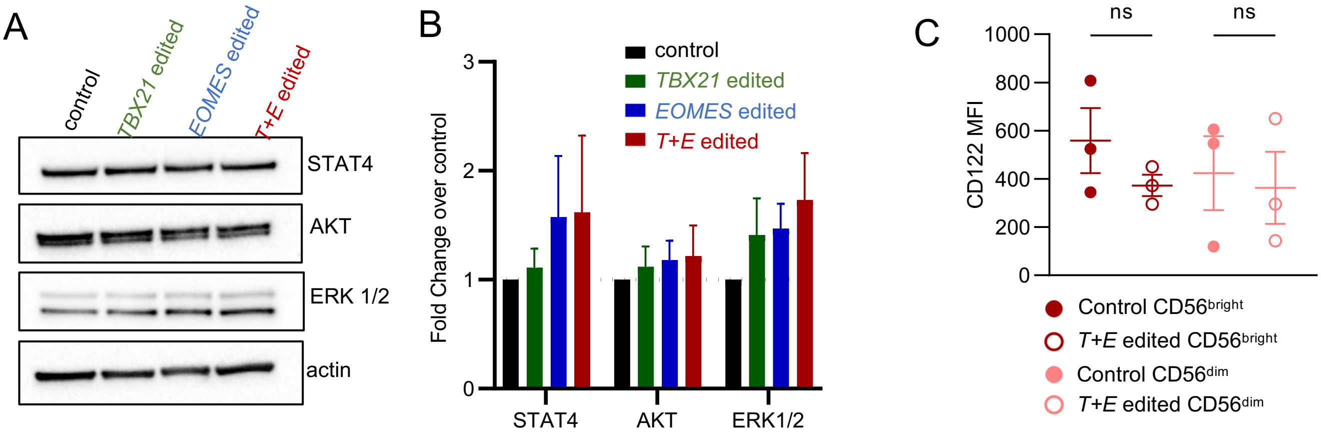

Supplemental Figure 5. Total signaling proteins downstream of IL-15 and IL-15 receptor subunit CD122 protein expression are not affected in *T+E* edited NK cells. (A) Representative western blot and (B) summary data of total STAT4, AKT, and ERK1/2 proteins within CRISPR-edited samples 1 week after electroporation. n=4 donors from 4 independent experiments. No statistical significance was found by 2-way ANOVA with Holm-Šídák multiple comparison test. (C) Expression of CD122 6 days after electroporation of NK cells cultured vitro. n = 3 donors from 3 independent experiments. No statistical significance was found by t-test.

Supplemental Figure 6

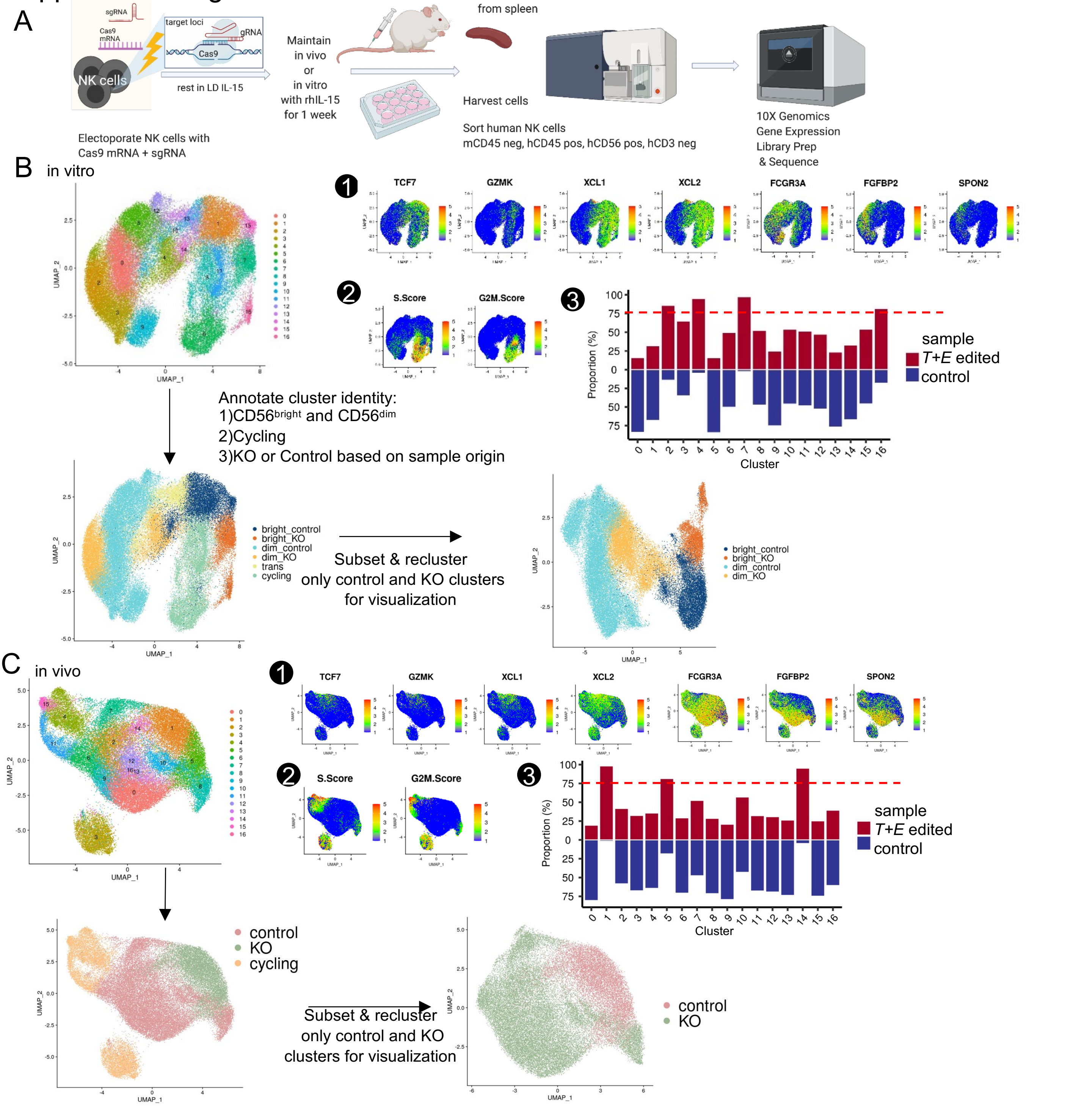

Supplemental Figure 6. UMAP plots of *TRAC*-edited(control) and *T+E* edited scRNA-seq samples show distinct clusters. (A) Schema of single cell RNA-sequencing experiment. The day after electroporation, *TRAC*-edited (control) or *T+E* edited NK cells were injected into NSG mice or maintained in vitro. After 1 week, splenocytes isolated from NSG mice and in vitro maintained NK cell were FACS sorted (live,hCD45+mCD45-CD56+CD3-). (B) UMAP of in vitro and (C) in vivo maintained samples. Clusters were first assigned to be CD56^{bright} or CD56^{dim} clusters based on known markers for each respective subset(CD56^{bright}:TCF7, GZMK, XCL1, XCL2; CD56^{dim}: FCGR3A, FCGBP2, SPON2). Cycling clusters were them identified by G2M and S scores. Then clusters were grouped into the following-"KO": non-cycling clusters where >75% of cells within the cluster originate from a *T+E* edited samples, "Control": non-cycling clusters where <75% of cells are T+E edited samples. Finally, "Control" and "KO" clusters were reclustered for visualization. n=5 donors, 5 independent experiments in vitro and n=3 , 3 independent experiments in vivo.

Supplemental Figure 7

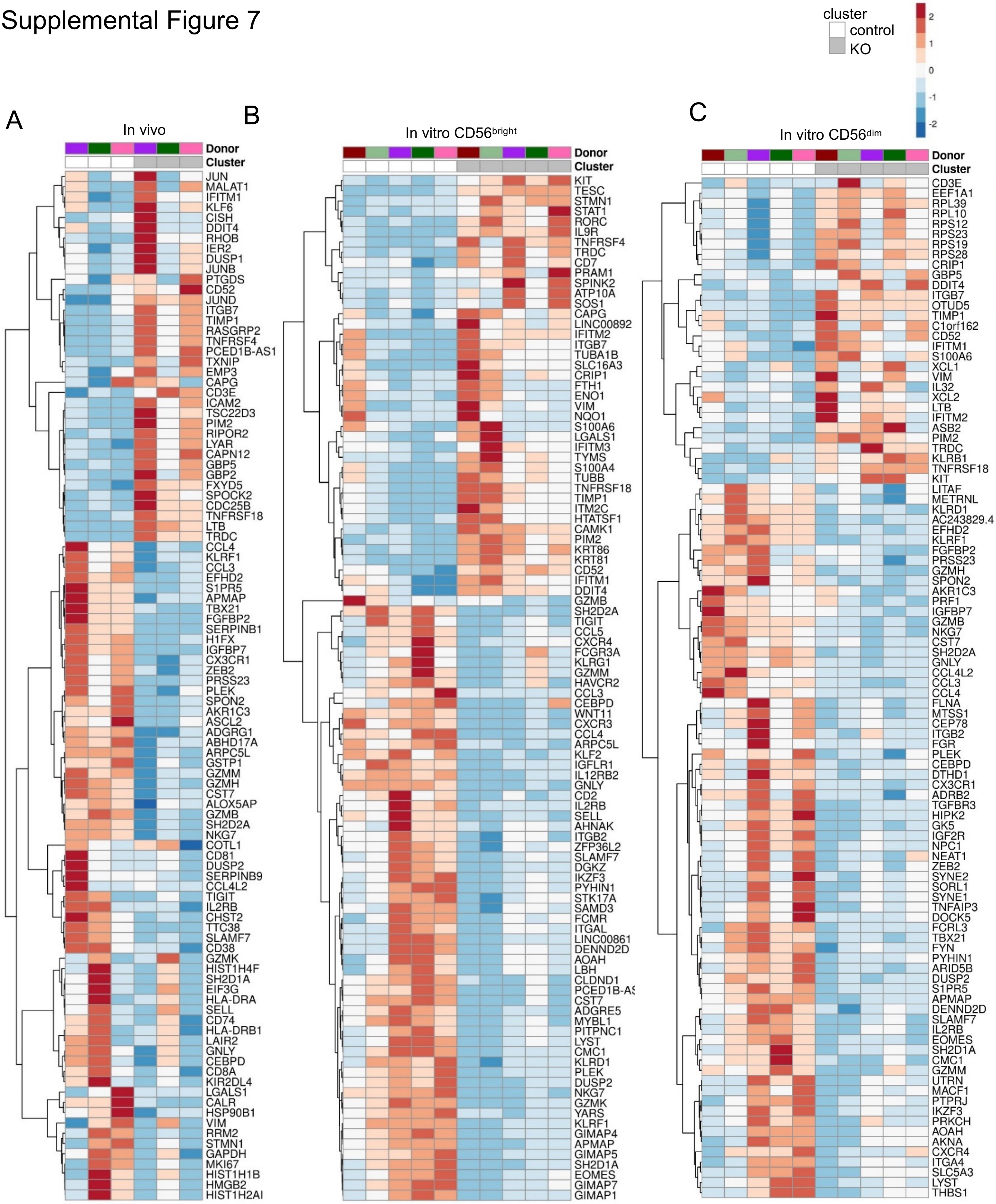

Supplemental Figure 7. scRNA-seq reveals transcriptional profile regulated by T-BET and EOMES in human NK cells. Heatmaps of average expression of Top 100(by Fold Change) differentially expressed genes of KO vs control clusters from (A) in vivo, (B) in vitro $CD56^{bright}$, and (C) in vitro $CD56^{dim}$ NK cells. n=5 donors, 5 independent experiments in vitro and n=3, 3 independent experiments in vivo.

Supplemental Figure 8

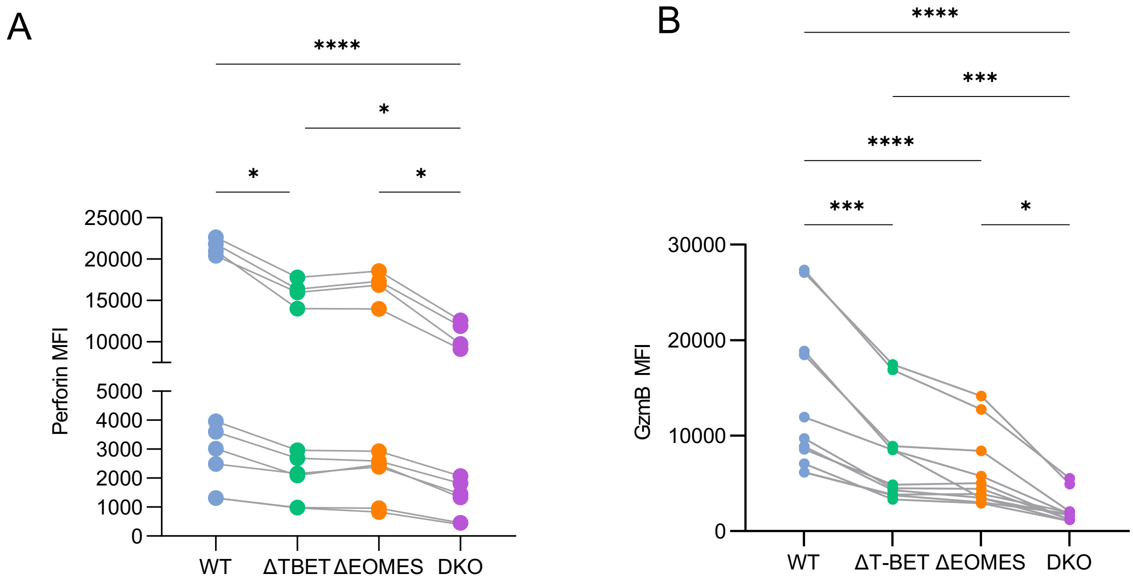

Supplemental Figure 8. T-BET and EOMES regulate protein expression of NK cell cytotoxic effector molecules. Expression of (A) PERFORIN and (B) GRANZYME B in *T+E* edited NK cells from spleens of NSG mice 1 week post NK cell injection. Data were compared using one way ANOVA with Tukey's multiple comparison test. n=5-6 donors, 10-11 mice total, 5 independent experiments.

Supplemental Figure 9

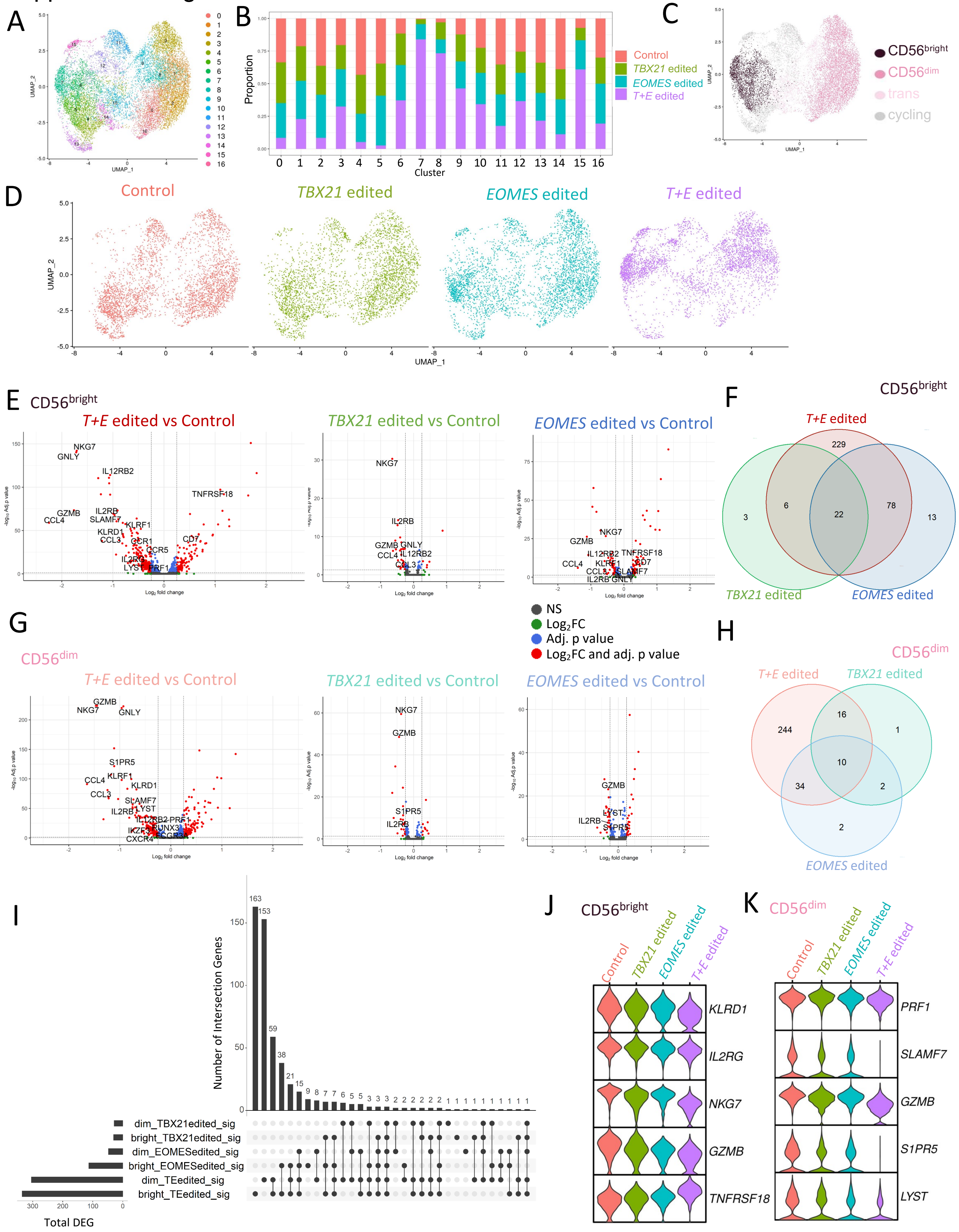

Supplemental Figure 9. T-BET and EOMES single knock-out have minimal effect on human NK cell transcriptional profiles. 8 days after CRISPR electroporation, NK cells were FACS sorted (live,hCD45+mCD45-CD56+CD3-) and subjected to single cell RNA-sequencing (10X Genomics). (A) UMAP of control, *TBX21* edited, *EOMES* edited, and *T+E* edited NK cells. (B) Distribution of sample groups in each cluster identified by FindClusters function in Seurat. (C) UMAP indicating seurat clusters assigned to be CD56^{bright} , transitioning, CD56^{dim} , and cycling based on known markers for each respective subset(Fig. S5) (D) Splited UMAPs showing control, *TBX21* edited, *EOMES* edited, and *T+E* edited samples separately. (E-K) Differential expression analysis was performed within CD56^{bright} or CD56^{dim} clusters. T*BX21* edited, *EOMES* edited, or *T+E* edited NK cells were compared against control samples within the respective clusters (E) Volcano plots of DEGs($|log2FC| > 0.25$, adj. p vale < 0.05), and (F) venn diagram depicting unique and overlapping DEGs in comparisons within CD56^{bright} cell clusters. (G) Volcano plots of DEGs and (H) venn diagram depicting unique and overlapping DEGs in comparisons within CD56^{dim} cell clusters. (I) Upset plot depicting intersections of genes with all 6 comparisons in CD56^{bright} and CD56^{dim} clusters. (J-K) Violin plots of example DEGs. n=2 donors from 2 independent experiments.

Supplemental Figure 10

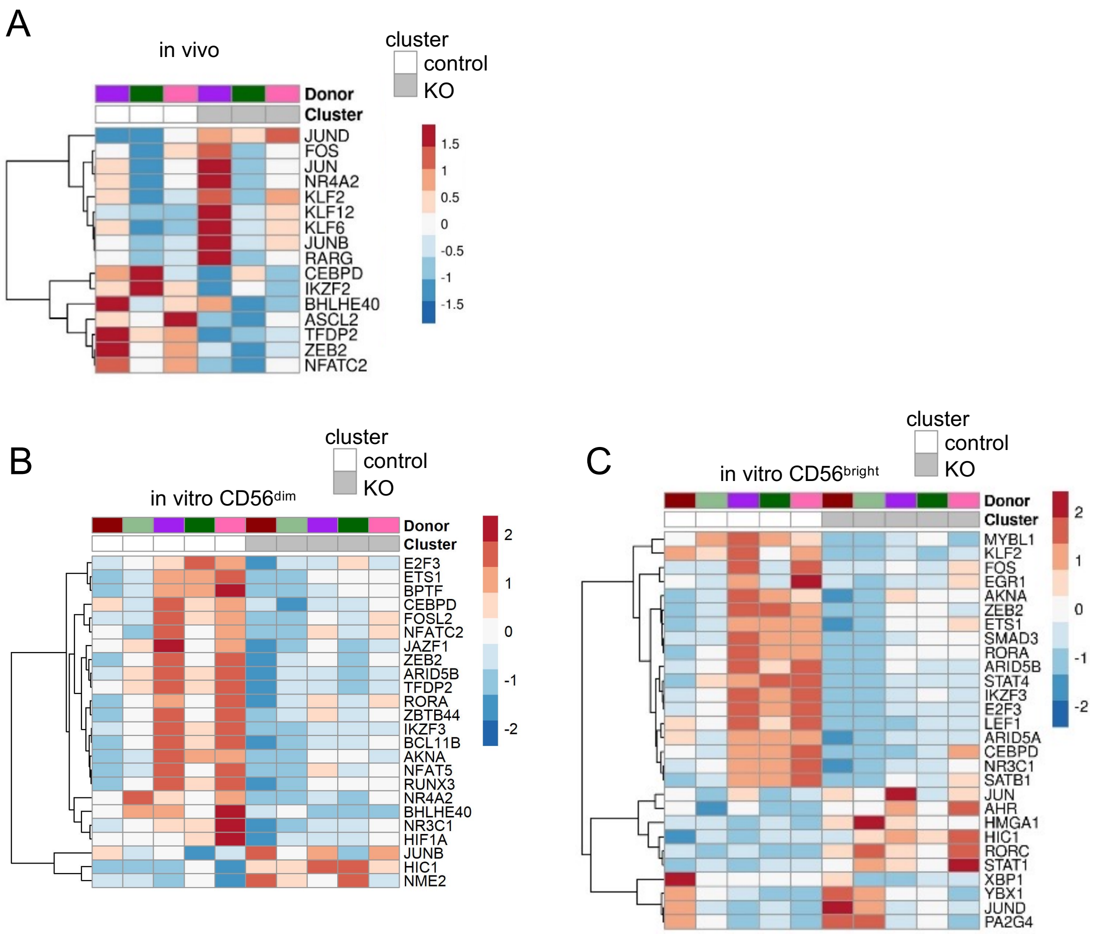

Supplemental Figure 10. T-BET and EOMES regulate expression of other transcription factors. Heatmaps of average expression of transcription factor genes in control and KO clusters of (A) in vivo, (B) in vitro CD56^{dim}, (C) in vitro CD56^{bright} NK cells in scRNA-seq experiment of control and *T+E* edited NK cells.
